# Drug design and repurposing with DockThor-VS web server focusing on SARS-CoV-2 therapeutic targets and their non-synonym variants

**DOI:** 10.1038/s41598-021-84700-0

**Published:** 2021-03-10

**Authors:** Isabella A. Guedes, Leon S. C. Costa, Karina B. dos Santos, Ana L. M. Karl, Gregório K. Rocha, Iury M. Teixeira, Marcelo M. Galheigo, Vivian Medeiros, Eduardo Krempser, Fábio L. Custódio, Helio J. C. Barbosa, Marisa F. Nicolás, Laurent E. Dardenne

**Affiliations:** 1 Grupo de Modelagem Molecular em Sistemas Biológicos (GMMSB), National Laboratory for Scientific Computing - LNCC, Petrópolis, RJ Brazil; 2 Instituto Federal Fluminense - IFF, Macaé, RJ Brazil; 3 Fundação Oswaldo Cruz - Fiocruz, Rio de Janeiro, RJ Brazil; 4 Laboratório de Bioinformática (Labinfo), National Laboratory for Scientific Computing - LNCC, Petrópolis, RJ Brazil

**Keywords:** Computational platforms and environments, High-throughput screening, Virtual drug screening, Structure-based drug design

## Abstract

The COVID-19 caused by the SARS-CoV-2 virus was declared a pandemic disease in March 2020 by the World Health Organization (WHO). Structure-Based Drug Design strategies based on docking methodologies have been widely used for both new drug development and drug repurposing to find effective treatments against this disease. In this work, we present the developments implemented in the DockThor-VS web server to provide a virtual screening (VS) platform with curated structures of potential therapeutic targets from SARS-CoV-2 incorporating genetic information regarding relevant non-synonymous variations. The web server facilitates repurposing VS experiments providing curated libraries of currently available drugs on the market. At present, DockThor-VS provides ready-for-docking 3D structures for wild type and selected mutations for Nsp3 (papain-like, PLpro domain), Nsp5 (Mpro, 3CLpro), Nsp12 (RdRp), Nsp15 (NendoU), N protein, and Spike. We performed VS experiments of FDA-approved drugs considering the therapeutic targets available at the web server to assess the impact of considering different structures and mutations to identify possible new treatments of SARS-CoV-2 infections. The DockThor-VS is freely available at www.dockthor.lncc.br.

## Introduction

Pandemic COVID-19 caused by the infection of severe acute respiratory syndrome (SARS) coronavirus 2 (SARS-CoV-2), initially described near the end of 2019^1^, left the world in lockdown and reached almost 35.6 million confirmed cases including 1,042,798 deaths by September 2020 (WHO Coronavirus Disease (COVID-19) Dashboard, 06 September 2020 https://covid19.who.int). SARS-CoV-2 belongs to the genus Betacoronavirus of the subfamily Coronavirinae, which includes other coronaviruses (CoVs), such as those responsible for the outbreaks of the severe acute respiratory syndrome (SARS-CoV) in 2002/2003 and the Middle East respiratory syndrome (MERS-CoV) in 2012^1,2^. The emergence of these CoVs, including SARS-CoV-2, demonstrated versatile host ranges and tissue tropism of these pathogens that can infect the respiratory, gastrointestinal, hepatic, and central nervous systems of humans, birds, bats, pangolins, and many other wild animals^3^.

The CoVs genome is a single-stranded positive-sense RNA (+ ssRNA) of approximately 30 Kb with 5′-cap and 3′-poly-A tail^4^. The SARS-CoV-2 genomic sequence is more similar (96.2%) to bat SARS (SARSr-CoV; RaTG13)^5^ than to human SARS-CoV (about 79%) or MERS-CoV (about 50%)^6^. However, SARS-CoV-2 uses the same receptor on the eukaryotic membrane surface as SARS-CoV, the angiotensin II-converting enzyme (ACE2)^7^. Although a new Spike-mediated CD147 receptor viral invasion route has recently been demonstrated for SARS-CoV-2, mediating viral invasion and disseminating virus among other cells^8,9^.

Structurally, the SARS-CoV-2 genome is organised in the order of the 5′-replicase polyprotein (ORF1/ab)-structural proteins [Spike (S)-Envelope (E)-Membrane (M)-Nucleocapsid (N)]-3′. There is a frameshift of -1 between ORF1a and ORF1b, leading to the production of two polypeptides: pp1a and pp1ab. Also, these polypeptides are processed into 16 non-structural proteins (Nsps, Nsp1‐16), whose cleavage is mediated by the Mpro (Nsp5, 3CLpro, or main protease) and a papain-like protease (Nsp3, PLpro)^10^. Other ORFs in a third of the genome near the 3′ terminal encode four main structural proteins: Spike, membrane, envelope, and nucleocapsid proteins. The new CoV encodes accessory structural proteins, namely ORF3a, 6, 7a/b, and ORF8, altogether totalling 29 proteins. All structural proteins are translated from viral subgenomic messenger RNAs (sgRNAs) produced by the replication and transcription complex (RTC), which includes both the RNA-dependent RNA polymerase (Nsp12, RdRp)^4,10^ and the 3′-5 'exoribonuclease with a functional proofreading-repair activity (Nsp14, ExoN)^4^.

Albeit their high copying fidelity, mutations in CoVs are observed as consequences of three known processes, namely (i) as lesions during the error-prone repair process; (ii) as a mechanism of RNA recombination and segment reassortment; (iii) by host-dependent RNA editing systems, such APOBECs and ADARs^11^. These genetic processes give rise to clouds of intra-host variants, according to the viral quasispecies dynamics^12^. Consequently, during the pandemic outbreak, the generations of polymorphic viral quasispecies can promptly arise interacting within the host, allowing viral immune evasion, resistance to antiviral drugs, as well as affecting the sensitivity of molecular diagnostic tests. Therefore, the monitoring of genomic changes in SARS-CoV-2 for identifying regions associated with drug resistance and vaccine evasion is essential in designing antiviral therapies.

There is a global effort from academic and non-academic groups to evaluate and develop an effective treatment for COVID-19. Many drug repositioning studies and compound evaluation to develop new antiviral drugs against SARS-CoV-2 have been developed using experimental^13–15^ and theoretical/computational^16–20^ approaches. More specifically, there is a vast interest in using high-throughput virtual screening approaches using different molecular modelling methodologies to investigate drug repositioning libraries and a plethora of compounds databases focusing on distinct SARS-CoV-2 molecular targets. Such computational studies often serve as a basis for further in vitro and in vivo studies. However, the success of virtual screening experiments depends on factors that are often not trivial to be addressed by non-specialist researchers^21,22^. Among the main factors are: (i) the correct choice and preparation of the molecular target structure (*e.g.,* protonation states of ionizable residues, incorrect side-chain conformations); (ii) proper preparation of the compound libraries (*e.g.,* pH-dependent protonation states, tautomerism, isomerism); (iii) receptor flexibility and, especially, induced-fit effects due to ligand binding; (iv) ligand flexibility (especially for peptides and macrocycle containing molecules); (v) performance of particular docking programs and scoring functions; and (vi) availability of computational resources for high-throughput virtual screening experiments. Moreover, possible genomic variations in the active/binding site region of molecular targets can drastically affect the binding mode and affinity of ligands and, consequently, change the identification of promising compounds.

Nowadays, the fast-increasing amount of available structural and genomic data of SARS-CoV-2 protein targets enhances the success of virtual screening and molecular modelling experiments. Herein, we adhere to research groups worldwide to enhance both new antiviral and drug repurposing research to develop an effective treatment for COVID-19. Our focus is on improving the DockThor-VS web server as a virtual screening platform to monitor the emergence of relevant non-synonymous mutations on SARS-CoV-2 target proteins. The DockThor-VS web server is freely available for the scientific community since 2013 as a docking platform for drug discovery containing the main steps of protein, cofactor, and ligand preparation, being able to deal efficiently with a wide diversity of protein–ligand systems for both binding mode and affinity prediction.

Herein, we present the developments implemented in the DockThor-VS web server as an effort to provide for the scientific community the possibility to perform COVID-19-related virtual screening experiments with: (1) curated structures of potential therapeutic targets from SARS-CoV-2 Nsp3 (papain-like, PLpro domain), Nsp5 (Mpro, 3CLpro), Nsp12 (RdRp), Nsp15 (NendoU), Nucleocapsid phosphoprotein (N) and Spike considering wild types and selected variants, and (2) curated libraries of the currently available drugs on the market.

To the best of our knowledge, the DockThor-VS platform is the unique virtual screening server that provides the users with curated datasets of both wild type and relevant mutants from SARS-CoV-2 therapeutic targets and prepared datasets for drug repurposing.

Besides considering relevant protein target mutations, our approach differs from other platforms mentioned in the literature^23,24^ by accounting for the following relevant aspects (cited above) when performing a virtual screening experiment. (i) For each protein target, we chose a set of structures representing different relevant conformations complexed with varying types of ligand (e.g., ligands covalently bound or not, side chains conformations closing or opening the binding site), thus providing an ensemble of representative structures of the flexibility of the receptor. (ii) For each protein target, the protonation states of ionisable residues were carefully investigated in the available literature. (iii) The repurposing libraries open to the users were carefully prepared considering the different protonation states and tautomerism for each compound. (iv) The DockThor docking program^25^ was specially developed to deal with highly flexible ligands, and it is very suitable to dock highly flexible peptides^26^. (v) Macrocycle drugs were subjected to special treatment to generate distinct conformations. (vi) The availability of the dataset of SARS-CoV-2 therapeutic targets allows the users to perform target fishing projects to search for the most promising target for the investigated compounds.

The DockThor-VS platform (freely available at https://www.dockthor.lncc.br) is coupled to the SDumont Brazilian supercomputer platform, supporting virtual screening experiments with up to 200 compounds for guest users and 5,000 compounds for registered projects.

In the next sections, we will show the technical details of the target selection and generation of the compound libraries and some general features of the DockThor-VS platform. We reveal and discuss the results of repurposing virtual screening experiments, considering the six therapeutic targets. We also assess the impact of considering different protein structures and mutations to identify possible new lead compounds to treat SARS-CoV-2 infections.

## Results and discussion

### Dataset of potential therapeutic targets

#### Non-structural protein 3 (Nsp3, papain-like protease—PLpro)

The multi-domain non-structural protein 3 (Nsp3) is the largest protein produced by the coronavirus, comprising 16 different domains and regions that regulate viral infection, with the papain-like protease domain (PLpro) being the most widely targeted domain from Nsp3. Since the outbreaks of SARS-CoV in 2003 and MERS-CoV in 2012, the three-dimensional structure of Nsp3 has been solved by X-ray crystallography and nuclear magnetic resonance (NMR) spectroscopy. Currently, we provide in the DockThor-VS web server monomeric structures and genomic variations of the PLpro domain. Structural information and selected mutations regarding the Macrodomain I/II/III (MacI/II/III) or active ADP-ribose-100-phosphatase domain (ADRP, app-1″-pase) will be available soon.

PLpro is a cysteine protease that processes, through self-catalyzed cleavage reaction, the amino-terminal end of the replicase polyprotein (pp1a) generating mature Nsp1, Nsp2 and Nsp3 proteins^27^. This protein is also responsible for aiding the coronavirus in its invasion by counteracting host innate immunity. The PLpro is a multifunctional enzyme capable of cleaving the viral polyprotein, and also functions as a deubiquitinase (DUB) and deISGylating (deconjugating interferon-stimulated gene 15 [ISG15] molecule from modified substrates), using identical catalytic residues^28^. Thus, the therapeutic inhibition of PLpro would have two antiviral effects: restoration of the antiviral effect of deubiquitinylation/ISGylation and inhibition of viral replication by blocking polyprotein cleavage^29^.

The SARS-CoV-2 PLpro catalytic site is composed of a classic triad Cys111-His272-Asp286. Cys111 performs the nucleophilic attack on the peptidic substrate, while His272 and Asp286 act by stabilizing the intermediate of the reaction^27^. Trp106 forms the oxyanion site participating in the stabilization of the negatively charged intermediate. Many non-covalent inhibitors interact at an allosteric site near the catalytic site. This allosteric site is mainly composed of the Asn267-Tyr268-Gln269 residues, forming a β-turn secondary structure^27^. Its flexibility is well described in the literature and is mainly characterized by distinct conformations of the residue Tyr268, which usually makes stacking interactions with aromatic groups of some inhibitors. The protonation state of the catalytic triad residues was defined based on the mechanism of reaction proposed in the literature for SARS-CoV^27^: neutral Cys111, His272 neutral at NE2 and Asp286 negatively charged.

**Figure 1:**
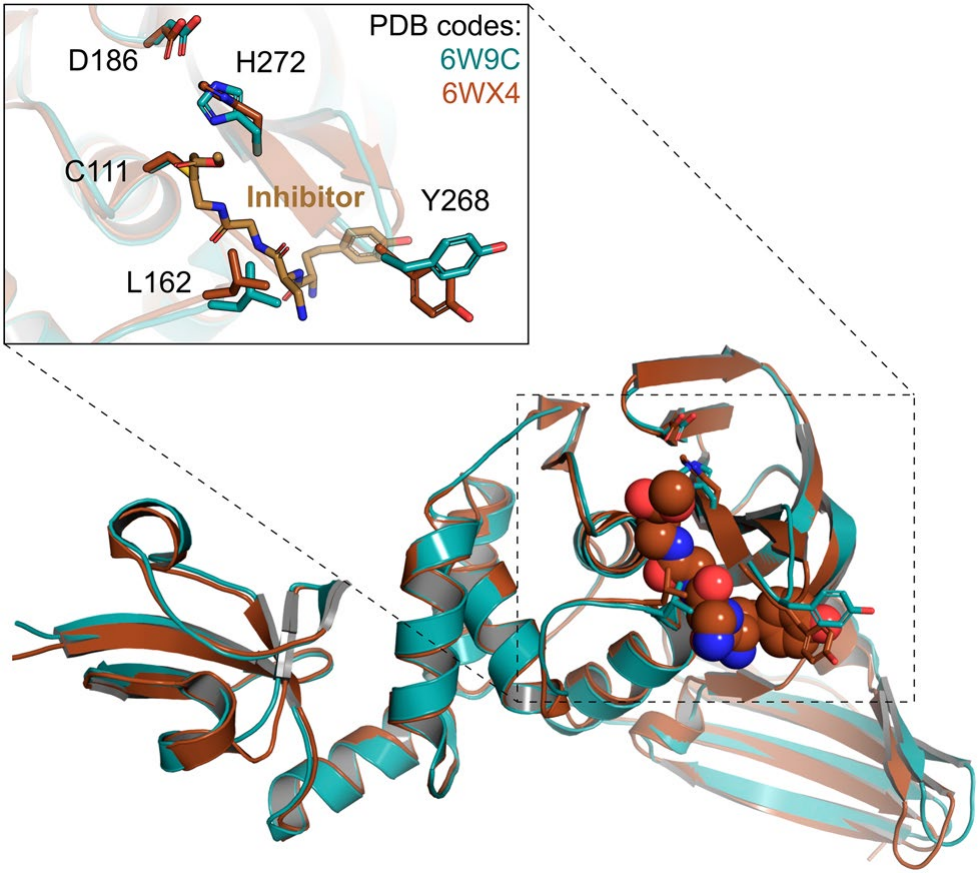
Conformations of the Nsp3-PLpro domain provided at the DockThor-VS web server as representative structures of the protein flexibility. The apo structure is coloured blue (PDB code 6W9C) and the structure initially complexed with a covalently bound peptide inhibitor is coloured brown (PDB code 6WX4^29^).

To date, there are 16 PLpro crystal structures in the PDB. Given the flexibility observed for Tyr268 from the allosteric site and Leu162, located at the entrance of the catalytic site, we provide to the users two prepared structures related with the PDB codes 6W9C (apo structure) and 6WX4^29^ (solved in complex with a covalently bound peptide inhibitor) (Fig. 1). In both structures, the Tyr268 is presented on an open conformation, allowing the binding of ligands with different sizes. The recently solved structure of PLpro complexed with a non-covalently bound compound (PDB code 7JIW) will be provided soon in the DockThor-VS.

#### Non-structural protein 5 (Nsp5, Mpro, 3CLpro)

As is well-known in coronaviruses, the two overlapping polyproteins pp1a and pp1ab, firstly produced after infection, are further proteolytically processed into 16 non-structural proteins (Nsp1–16). This proteolytic process is carried out in a coordinated manner by the PLpro and the Mpro^30^. Mpro is also known as 3-chymotrypsin-like cysteine protease (CCP or 3CLpro), that first is auto-cleaved from polyprotein pp1a to yield the mature enzyme and then digests the remaining pp1a (at least by 11 conserved sites) to produce the downstream non-structural proteins (Nsps 6 to 16)^31^. Given the pivotal role of Mpro in the viral life cycle, it becomes an attractive target for the design of anti-SARS drugs.

**Figure 2:**
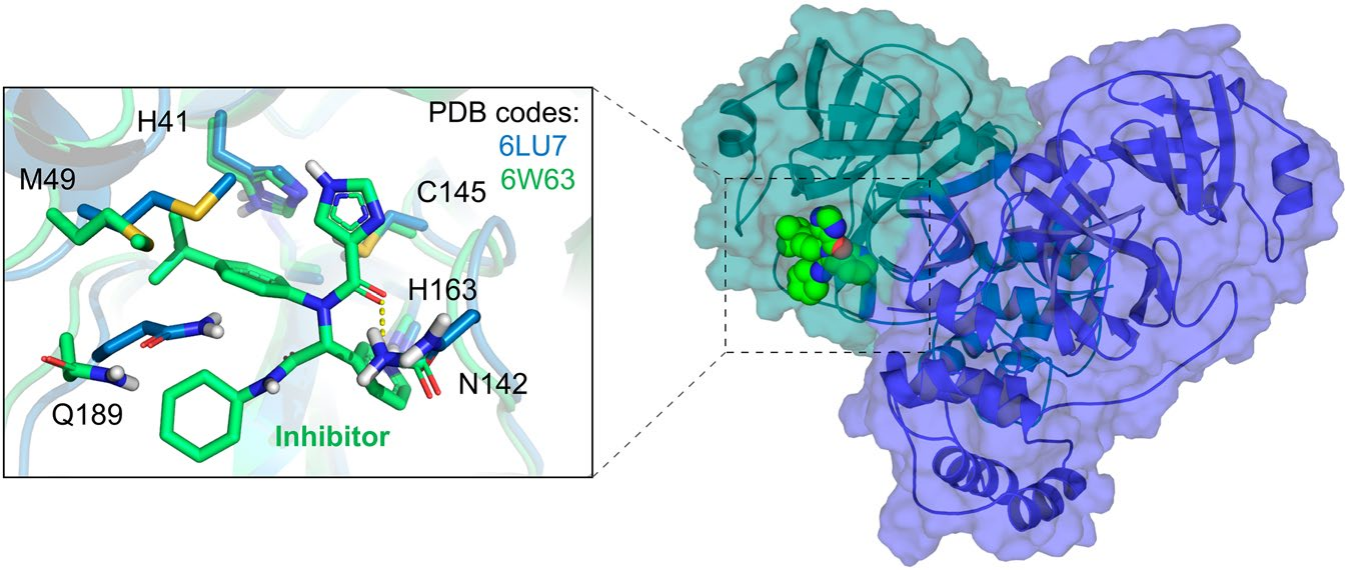
Conformations of the Mpro provided at the DockThor-VS web server as representative structures of the protein flexibility. The protein complexed structure with a covalent inhibitor is coloured blue (PDB code 6LU7, the ligand is not shown) and the structure initially complexed with a non-covalent inhibitor is coloured green (PDB code 6W63). The protein–ligand hydrogen bonds are represented as yellow dashed lines.

The Mpro consists of a homodimer with each polypeptide composed of three domains: I (residues 8–101), II (residues 102–184) and III (residues 201–303). The substrate-binding site is located in a cleft between the domains I and II. It has the Cys145-His164 catalytic dyad as the reaction center, following a mechanism similar to other coronaviruses. There are currently 191 structures of the Mpro deposited in the PDB, with many of them complexed with covalent or non-covalent inhibitors. At this moment, we provide to the users the dimeric structure of the Mpro in two distinct conformations (PDB codes 6LU7^30^ and 6W63), which are complexed with covalent and non-covalent inhibitors, respectively. The structures’ superposition highlights some significant conformational changes within the ligand-binding site, mainly the residues Met49, Asn142 and Gln189. That reinforces the importance of considering multiple protein conformations in virtual screening experiments to accommodate distinct compounds (Fig. 2). While the 6LU7 conformation was the first structure experimentally solved for the SARS-CoV-2 Mpro with an inhibitor, the 6W63 contains a drug-like reversible inhibitor at the binding site. In the preparation process, the protonation states and flips of key residues were manually adjusted to provide the Mpro structures with neutral His41 at ND1, the catalytic Cys145 protonated (*i.e.,* neutral), neutral His163 at NE2 and neutral His164 at NE2.

#### Non-structural protein 12 (Nsp12, RdRp)

To replicate and transcript positive ssRNA, an RNA-dependent RNA polymerase (RdRp, also known as Nsp12) of coronaviruses has evolved to perform this process forming an intricate complex with several non-structural proteins (Nsps) produced as cleavage products of the ORF1a and ORF1ab viral polyproteins. Nsp12 catalyzes the synthesis of viral RNA and possibly with the assistance of Nsp7 and Nsp8 that function as cofactors^32^. In SARS-CoV-2 the overall architecture of the Nsp12-Nsp7-Nsp8 complex is similar to that of SARS-CoV with a root mean square deviation (RMSD) value of 0.82 for 1078 Ca atoms^33^.

RdRp is considered an interesting target for therapeutic solutions against COVID-19, for which the inhibitor remdesivir (RDV, GS-5734), a nucleoside analogue prodrug of the ebola virus (EBOV) RdRP has been already approved^34^. Since the nucleoside analogues have a high structural similarity, other similar drugs such as favipiravir, which was effective in clinical trials, can be used as an inhibitor^35^.

The conserved architecture of the Nsp12 core consists of a right-hand RdRp domain (residues Ser367 to Phe920) and a nidovirus-specific N-terminal extension domain (residues Asp60 to Arg249) that adopts a nidovirus RdRp-associated nucleotidyltransferase (NiRAN) architecture^33^. The average length of the core RdRp domain is less than 500 amino acids and is folded into three subdomains, namely thumb, palm, and fingers resembling a right-handed cup^36^. The NIRAN and RdRp domains are connected by an interface domain (residues Ala250 to Arg365). Besides, COVID-19 virus Nsp12 possesses a newly identified β-hairpin domain at its N terminus^33^.

The active site of the SARS-CoV-2 RdRp domain is formed by the conserved polymerase motifs A to G in the palm subdomain and configured like other RNA polymerases^33^. Remarkably, the motifs A and C have conserved residues characteristic of viruses + ssRNA, such as the catalytic aspartates in motifs A (Asp618) and C (Asp760)^37^. Motif B has highly conserved Ser682 that is crucial for recognizing the 2′-OH group of the NTP ribose and Gly683, which is conserved in all RdRps^37^. Motif D and motif A, both guide the structural change of the active site during catalysis^38^. Regarding the nucleotide (NTP) selection by RdRp, motif D has a prime role in NTP addition’s efficiency and fidelity. Indeed, NMR studies have indicated the inability of motif D to achieve its optimal conformation for catalysis when an incorrect nucleotide is incorporated, thereby demonstrating its role in selecting NTPs^39^. Motif E together with motif C interact (in the upstream position) with the newly synthesized backbone of the second and third nucleotides, motif F establishes the upper limit for the entry path of NTPs^37^. In the motif F of SARS-CoV-2, the residue Ala547 of the N-Terminal region is equivalent to the highly conserved glutamate in almost all + ssRNA viruses^37^. This amino acid change leads to structural and possibly dynamic differences in this region, which can interfere with the RNA synthesis^40^. The motif G uses the residues S96 and A97 that interact with residues + 1 and + 2 of the template ribbon’s backbone to move it vertically^37,41^.

**Figure 3:**
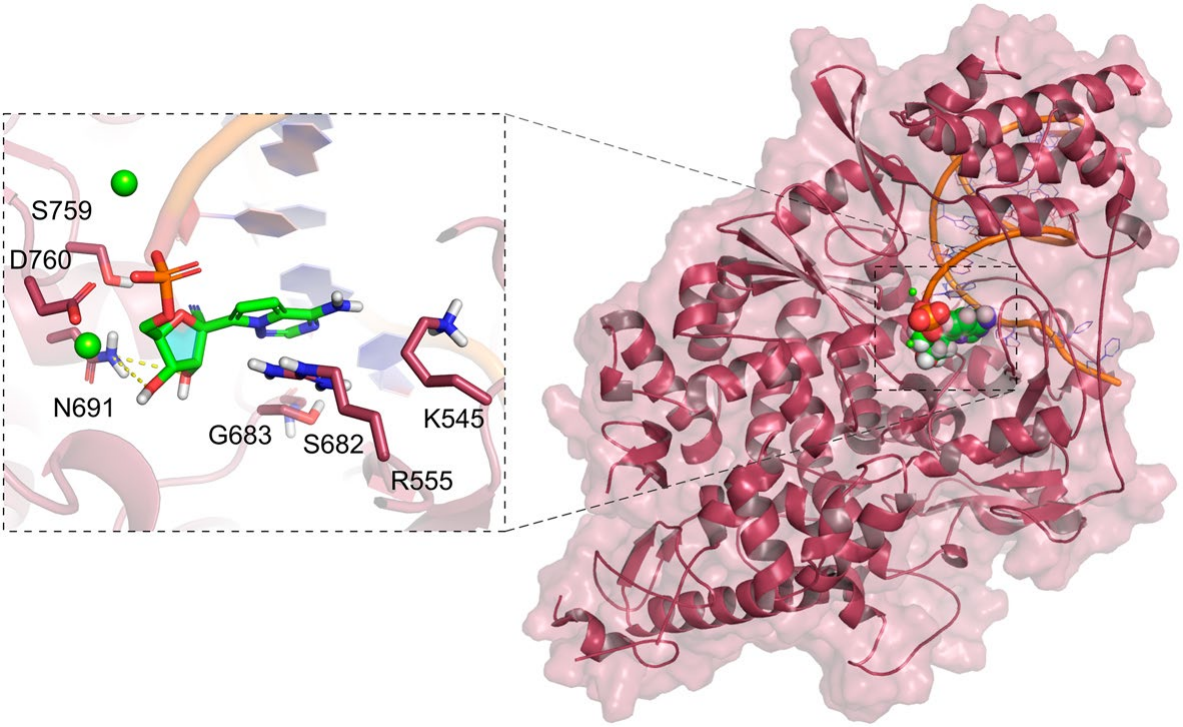
Structure of the Nsp12 complexed with remdesivir and an RNA primer (PDB code 7BV2, ligand coloured greed and RNA represented as an orange cartoon). Mg^2+^ ions are represented as green spheres. The protein–ligand hydrogen bonds are represented as yellow dashed lines.

To date, eight RdRp structures have been deposited at the PDB. We provided at the DockThor-VS platform the RdRp conformation found in the RdRp-RNA-remdesivir complex (PDB code 7BV2^42^, Fig. 3) without the RNA primer and the inhibitor remdesivir to allow the virtual screening experiments with the free binding site.

#### Non-structural protein 15 (Nsp15, endoribonuclease, NendoU)

The Nsp15 of SARS-CoV-2 is a nidoviral RNA uridylate-specific endoribonuclease (NendoU) that displays its RNA endonuclease activity (specific for uridine) acting on both, single-stranded RNA and double-stranded RNA^43^. Recently, Susan Baker’s Lab revealed for the first time, the molecular mechanism of Nsp15, in which the NendoU activity limits the generation of 5′-polyuridines from negative-sense viral RNA, termed PUN. The PUN can act as a CoVs MDA5-dependent pathogen-associated molecular pattern (PAMP), which in turn can activate the type I interferon (IFN) response in macrophages. The authors found that NendoU cleaves the polyU sequence on the PUN RNA, limiting the length and abundance of the polyU extension. These studies revealed that the function of NendoU during replication is to reduce the length of polyU sequences, thus limiting the potential to generate PAMPs and activate the host sensor MDA5. Consequently, the NendoU activity delays recognition by the host innate immune sensors, and therefore, Nsp15 is a highly conserved virulence factor and a potential target for antiviral and vaccine strategies^43^.

The Nsp15 endoribonuclease from SARS-CoV-2 comprises 347 amino acid residues (sequence from Met1 to Gln347)^44^. The SARS-CoV-2 Nsp15 monomers group into a functional hexamer, composed by a dimer of trimers^44^. The hexameric form is pivotal for the enzymatic activity. Each monomer presents three domains: (i) the N-terminal (Nsp15-NTD, residues 1–62), formed by an antiparallel β-sheet wrapped around two α-helices; (ii) the central middle (residues 63–191), composed by β-strands and short helices; and (iii) the C-terminal catalytic NendoU domain (NendoU, residues 192–347), formed by two antiparallel β-sheets. There are currently seven high-resolution crystal structures of Nsp15 endoribonuclease from SARS‐CoV‐2 available at the PDB containing the three domains.

**Figure 4:**
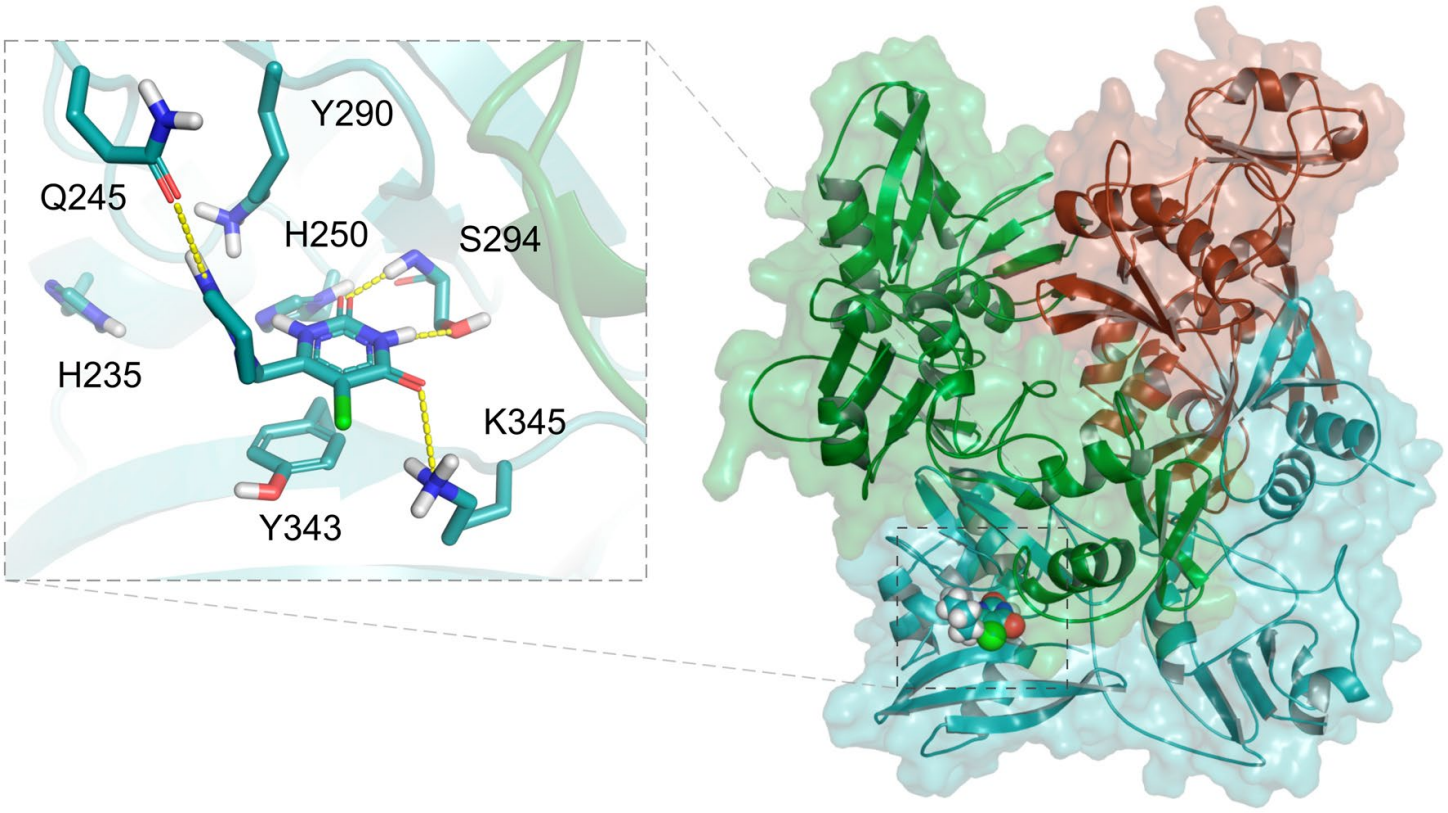
Structure of the Nsp15 trimer complexed with Tipiracil (PDB code 6WXC). The protein–ligand hydrogen bonds are represented as yellow dashed lines.

The active site is located at the CTD, flanked by five α-helices in its concave surface, in a shallow groove between two β sheets, and contains six highly conserved residues: His235, His250, Lys290, Thr341, Tyr343 and Ser294. Based on the similar arrangement of its active site with that of Ribonuclease A, the residues His235, His250 and Lys290 are suggested to be the catalytic triad of the NendoU^44^. The prepared Nsp15 structure is based on the conformation of the protein complexed with tipiracil (PDB code 6WXC) prepared considering the pH of 6.2 and consists of His235 and His250 neutral at NE2 and ND1, respectively, and Lys290 positively charged (Fig. 4). New Nsp15 conformations will be available soon at the DockThor-VS platform.

#### Nucleocapsid phosphoprotein (N protein)

The nucleocapsid protein has an essential structural function in CoVs. This target is a multifunctional phosphoprotein that establishes an arrangement with genomic RNA forming the ribonucleoprotein (RNP) complex and plays a critical role during transcription, virus assembly and antagonism of host’s innate immunity. The N protein can form a helical filament structure that is assembled into virions by interactions with the viral membrane (M) protein^45^. Despite its location within the virion rather than on its surface, N protein is highly immunogenic and abundantly expressed during viral infection^46^. Interestingly, it has been demonstrated that the antibody to the SARS-CoV-2 N protein is more sensitive than the Spike protein antibody during the early infection^47^. Regarding the context of viral infection, the nucleocapsid protein acts as a viral suppressor of RNAi (VSRs), and thereby antagonizes one of the cell-intrinsic antiviral immune defence mechanisms of the host^48^. Mainly, during RNA viral infection, virus-derivated dsRNA (vi-dsRNA) are generated, which could be recognised and cleaved by the host endonuclease Dicer into virus-derived siRNAs (vsiRNAs). These vsiRNAs ultimates are integrated into de Argonaute protein within the RNA-induced silencing complex (RISC) directing the destruction of cognate viral RNAs in infected cells^49^. Jingfang Mu and collaborators (2020) showed that nucleocapsid of SARS-CoV-2 associates with dsRNA and suppresses RNAi by sequestrating viral dsRNA in cells, which probably prevents its recognition and cleavage by the host endonuclease Dicer^48^. Therefore, the N protein also represents a prime immune evasion factor of SARS-CoV-2, contributing to the pathogenicity of this new coronavirus. Consequently, the nucleocapsid phosphoprotein can be an attractive target, for example, to inhibit the viral life cycle stages, or else to recover the host's immunity mediated by an antiviral RNAi system.

The structure of N protein from coronavirus is composed of three domains: N-terminal RNA binding (N-NTD), C-terminal dimerisation (N-CTD) and central Ser/Arg (SR)-rich linker^50^. At the time of writing of this manuscript, there are 12 experimentally solved structures for the N protein, where three of them are related to the N-NTD. We provide to the users experimentally solved structures of the N-NTD already prepared for docking. The preparation of the N-CTD will be available soon. Currently, there are no drugs or potential compounds experimentally validated as SARS-CoV-2 N protein inhibitors.

Herein, we provide five monomeric structures of N-NTD obtained by NMR experiments (PDB code 6YI3) to account for the protein flexibility. Specifically, we depicted the basic finger moiety, which is commonly locked in one conformation in the X-ray solved structures available due to crystal lattice contacts^51^. According to studies with the N protein from the Influenza virus, antiviral drugs targeting N proteins should stabilise the monomeric form or induce abnormal oligomerization or interfere with the RNA binding^52^. Also, they suggested that the monomeric form binds to the replicating viral RNA in infected cells.

Recently, surface plasmon resonance (SPR) analysis experiments of SARS-CoV-2 N-NTD show low binding affinities for different ribonucleotide (AMP/UMP/CMP), except GMP, suggesting potential distinct ribonucleotide-binding mechanism between SARS-CoV-2 and HCoV-OC43 N protein^50^. Some important characteristics observed in the experimentally solved structures of SARS-CoV-2 N-NTD protein that may explain these findings are: (i) the N-terminal tail is highly flexible and adopts more opened conformations than in HCoV, probably allowing the interaction with viral RNA genome of high order structure, (ii) replacement of Tyr102 in HCoV to Arg89 located near to the nitrogenous base recognition site, (iii) phosphate-binding site containing Thr54 and Ala55 in SARS-CoV-2 instead of Ser67 and Gly68 in HCoV.

According to NMR-based titration experiments of N-NTD with a short double-stranded RNA (5′-CACUGAC-3′ and 5′-GUCAGUG-3′), the amino acid residues Ala50, Thr57, His59, Arg92, Ile94, Ser105, Arg107, Arg149, Tyr172 were proposed to form the molecular interface of the N-NTD: RNA complex^51^. Curiously, some critical residues involved in RNA recognition on other CoV N proteins such as HCoV, Tyr109 and Tyr111, were not affected by the RNA binding in the NMR titration experiments. However, they are well conserved among the coronavirus and remain to occupy the same spatial region in the SARS-CoV-2 structures compared to the HCoV-OC43 structure (PDB code 4LI4^53^).

**Figure 5:**
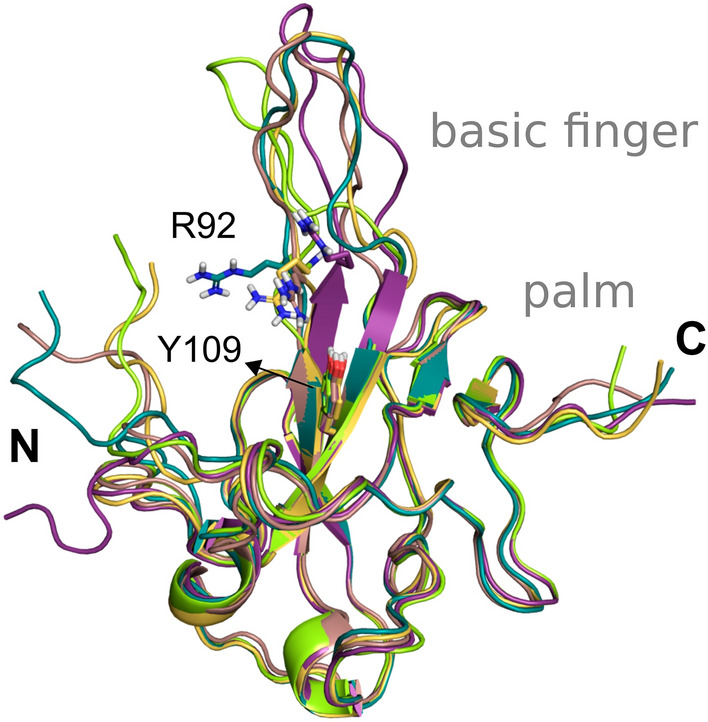
States from the NMR spectroscopy selected as the five representative structures of the N-NTD flexibility after clustering based on the conformations of Arg92 and Tyr109.

Thus, we provide to the users five distinct conformations of the N-NTD solved with NMR after clustering the 31 conformations containing the Glu174 on an opened conformation and selecting representative structures according to the flexibility of the residues Arg102 and Tyr109 (Fig. 5). The suggested binding site for docking experiments is centred on the hotspot located at the surface of the N-NTD between the finger and palm subdomains, which have been claimed as essential for RNA binding and a target site for small molecules^51^.

#### Spike glycoprotein

Spike protein (S) is a class I virus fusion protein^54^, and it is the limiting factor for the virus to enter the host cell^55^ using the Angiotensin-Converting Enzyme 2 (ACE2) as the main receptor^56^.

Spike is a homotrimer in which each monomer is about 180 kDa and contains approximately 1,273 residues^57^. It consists of the N-Terminal-Domain (NTD) S1 subunit covering residues 1—667 and will direct the link with the receptor, and the C-Terminal-Domain (CTD) S2 subunit that covers residues 668—1,273 and will be responsible for the merger between the virus and host membranes^57^. The S1 subunit is the main target for the development of new drugs because it has a region responsible for the interface of interaction with the host receptor called Receptor Binding Domain (RBD). The RBD is between residues 319 and 541 and its region that performs direct contact with the host’s receptor is called Receptor Binding Motif (RBM) and is located between residues 437 and 508 (Uniprot ID P0DTC2)^58^.

By describing the conformation states "up" and "down" of Spike's S1 structure, it is possible to illustrate the states of interaction with the receptor^59^. In the “down” configuration, the receptor is in an inaccessible state, while in the “up” configuration the receptor is in an accessible state. Since the ACE2 receptor only interacts with the RBD when it is in the “up” conformation, the down conformation would leave the RBD inaccessible to ACE2 or even to any possible inhibitor on this interface^60^. For this reason, all the structures that we are providing to the users are in the “up” conformation.

**Figure 6:**
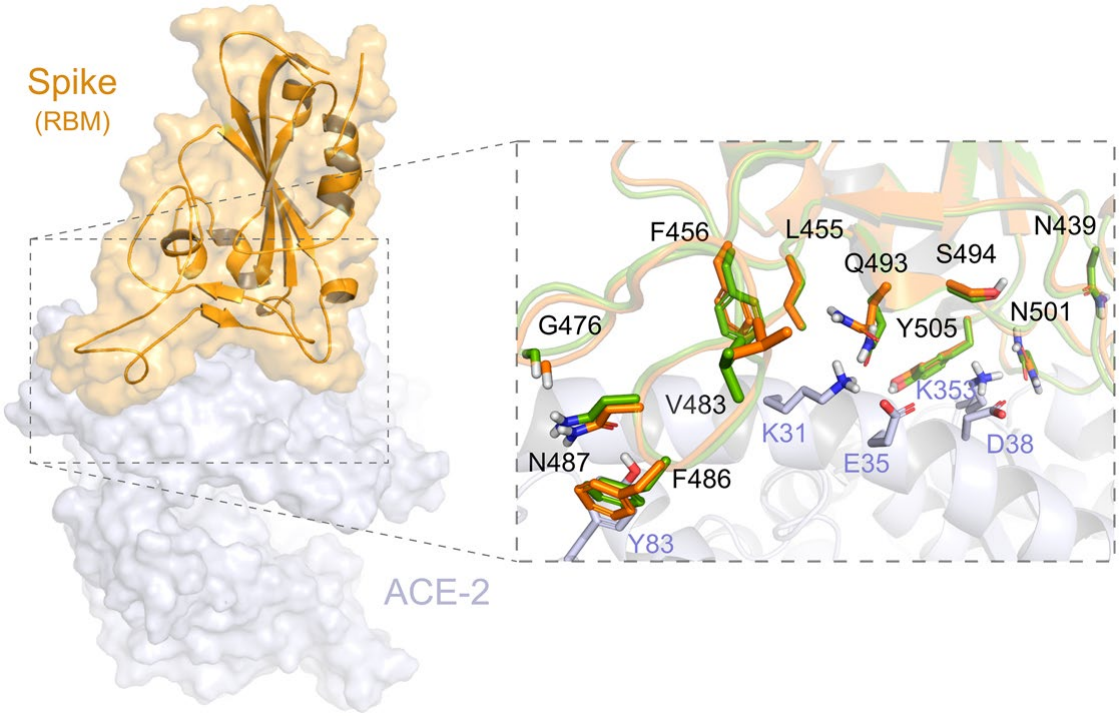
Conformations of Spike (RBM domain) available at the DockThor-VS platform. The human receptor ACE2 is coloured grey. The Spike-ACE2 interface is illustrated with zoom. The two Spike conformations (PDB codes 6M0J and 7BZ5) are coloured orange and green, respectively.

Until now, there are 69 structures available in the PDB related to the SARS-CoV-2 Spike protein, of which nine are complexed with ACE2, and four with neutralising antibodies. We currently provide at the DockThor-VS platform three Spike structures: the Spike-ACE2 complex and without ACE2 (PDB code 6M0J^58^). The Spike conformation found in the PDB code 7BZ5^61^ without the neutralizing antibody (Fig. 6).

In the preparation process, we kept the Asn and Gln flips predicted by the PrepWizard/PROPKA tool since some of them are part of the Spike protein–protein interaction interface and may be influenced by the interacting partner. For example, Gln493 and Asn501 were predicted with different flips when Spike is complexed with ACE2 (PDB code 6M0J) or the neutralizing antibody (PDB code 7BZ5).

### Non-synonymous variations in the selected targets

The ongoing pandemic spread of SARS-COV-2 resulted in the increasing generation of thousands of genome sequences (available in the GISAID repository, https://www.epicov.org, 376,417 sequences on 15/01/2021). Massive sequencing of SARS-CoV-2 genomes allows performing innumerable comparative, evolutionary and epidemiological analyses, as well as to identify the circulating genomic variants containing diverse genetic mutations, such as synonymous or non-synonymous variations (NSVs), deletions and nucleotide insertions. Notably, for the rational drug design, more attention is given to the study of NSVs in the coding regions since the substitution of amino acids can affect fold, binding affinity, post-translational modification, protein–protein interaction (PPI) and other protein characteristics^62^. Even so, some NSVs may not produce visible changes in the structure of the protein; in that case, the mutation may not have a biological impact (neutral). Alternatively, with the intra- and inter-host viral evolution in infected humans (quasispecies dynamics), the purifying selection can eliminate deleterious mutations over time, which are more detrimental to the pathogen's fitness, or else the positive selection promotes the spread of beneficial ones^63^.

**Figure 7:**
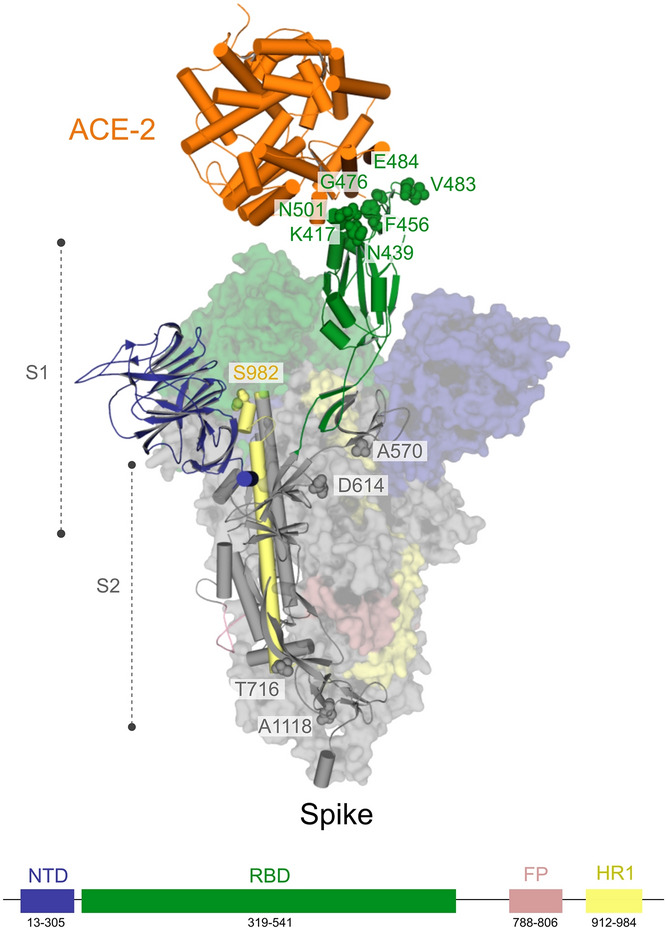
Position of Spike amino acid residues corresponding to the mutations highlighted in this study. Amino acid residues in the RBD (green) and outside of RBD (gray and light brown) mapped onto a structure of SARS-CoV-2 Spike determined by electron microscopy (PDB code 7DF4). For this analysis, only the mutations with those residues falling into RBD were considered. Critical residues outside of RBD related to substitutions: D614G, P681H, T716I, S982A, and D1118H, found in the variant VOC-202012/01, are shown.

The estimated mutation rate underlying the global diversity of SARS-CoV-2 is approximately 6 × 10^−4^ nucleotides/genome/year^64^, which is considered moderate for coronaviruses with the Nsp14 proofreading correction mechanism. Currently, the genomic analysis of almost 243 thousand circulating genomes from patient samples showed that there are more than 32 thousand replacements among 26 out of 29 proteins encoded on the SARS-CoV-2′s genomes in comparison with the reference genome sequence of isolate Wuhan-Hu-1 (NC_045512.2) (CoV-GLUE^65^, accessed on January 15, 2021). The distribution of this genomic diversity shows high allele frequency for five replacements in just four proteins, namely Spike (D614G, 91.28%, Fig. 7), Nsp12 (P323L, 91.04%), N (R203K, 36.81%, G204R, 36.55%) and ORF 3a (Q57H, 21.25%). The remainder corresponds to numerous NSVs with low alleles frequency (~ 11% to 0.002%). Ultimately, this can be explained by the positive selection that acts at a higher rate after the zoonotic transfer, suggesting an increasing mutant load in the circulating strains of SARS-CoV-2 in the epidemiological scenario^66^.

Considering 242,865 SARS-CoV-2 genomes, the total number of replacements per target selected for this study varied from 875 (Nsp5), 1,322 (Nsp15), 1,560 (N), 2,491 (Nsp12), 4,360 (Spike) up to 6,869 (Nsp3) (CoV-GLUE^65^, accessed on January 15, 2021). Here we selected a total of 19 NSVs covering the six selected targets (Table S1) with their variant structures already available to the users through the DockThor-VS web server. We assessed some parameters for the mutation’s choice, such as the impact of the residue’s occurrence in the catalytic region and its possible interaction with a ligand and the amino acid properties (hydropathy, charge and side chain). Alternatively, we also have considered the effect on the biological function (neutral vs deleterious) and searched in the literature any mutagenesis experiments with evidence for alteration in the protein’s molecular function or viral fitness in CoVs involving the focused residue.

For PLpro, we chose three amino acid substitutions with neutral functional effect, whose corresponding residues fall into the Peptidase C16 domain (Table S1). So far, we have selected only the replacement M165I on the Mpro, highlighting that the residue falls on a beta-sheet and is directly part of the ligand-binding site, with the side chains oriented towards the ligand (Table S1). Here, we describe one selected non-synonymous variation on the RdRp, namely G683V with deleterious functional effect (Table S1). Replacement G683V on the RdRp increases the volume of the side chain of a highly conserved glycine^37^, and it has already been described in vitro as a deleterious NSV^33^. We selected the four non-synonymous variations on the Nsp15, S293A, S293T (Ser294 in PDB 6VWW), Y342C and Y342H (Tyr343 in PDB 6VWW), whose residues are falling directly on the ligand interaction binding site. Particularly NSVs S293A and S293T are interesting since the Ser293 accounts as the critical residue for enzyme discrimination between uracil to cytosine or adenine to guanine bases^44^. For the nucleocapsid phosphoprotein, we selected the NSVs A50V, R92S and R149L, whose residues fall on the RNA-binding surface of its cognate domain^67^. These substitutions have a neutral (A50V and R92S) or deleterious (R149L) predicted functional effect (Table S1).

Finally, for the Spike glycoprotein of the SARS-CoV-2, we selected seven amino-acid substitutions (K417N, N439K, F456L, G476S, V483A, E484K and N501Y) (Table S1 and Fig. 7), whose residues are within the RBD (from residues 319 to 541) or RBM (from residues 437 to 508)^58^. The residues Phe456 and Asn439 are both critical for the interaction interface with the human receptor ACE2. Regarding Phe456, it is interesting to mention that a single amino acid substitution on the equivalent residue in SARS-CoV Spike glycoprotein (Leu443) affected both the antibody binding and neutralization^58^. Similarly, a mutagenesis assay in SARS-CoV on the equivalent residue of Asn439 (Arg426) demonstrated that at least two amino acid substitutions significantly reduced the binding to ACE2^68^. Concerning the residue Gly476, deletion mutagenesis of the equivalent positively charged region in the RBD of the SARS-CoV Spike (SΔ, 422–463) abolished the ability to induce potent neutralising antibodies in vivo as well as mediate viral entry^69^. On the other hand, studies with the equivalent position of the residue Val483 in MERS-CoV (Ile529) showed that single amino acid substitution reduced the host’s receptor affinity, with the consequent increase in resistance to antibody-mediated neutralization^70^. We further included three lineage-defining mutations in the spike protein with critical residues in the RBD: K417N, E484K and N501Y. The three amino acid substitutions are characteristic (but not unique) to the South Africa lineage B.1.351 (also known as 501Y.V2 variant), which emerged after the first epidemic wave in the worst affected metropolitan area within the Eastern Cape (EC) Province^71^. The N501Y mutation has also been identified in a new lineage in the United Kingdom: B.1.1.7 of Variant of Concern (VOC) designated VUI-202012/01 on the detection and re-designated as VOC-202012/01 on 18/12/20^72^. Asn501 forms a hydrogen-bonding interaction with Tyr41 in the binding loop region of ACE2, contributing to higher ACE2-binding affinity of SARS-CoV-2 than SARS-CoV-1^73,74^. A deep mutational scanning analysis showed that the N501Y replacement enhances binding affinity to ACE2, which was also demonstrated through in a mouse model^75,76^. While N501Y mutation may have a role in escaping class 1 neutralizing antibodies, Xie et al.^77^ found that antibodies generated by 20 participants with viruses carrying the N501Y mutation were roughly as potent as antibodies produced to combat Asn501-carrying viruses^71,77^. Lys417 forms a salt bridge with Asp30 of ACE2 across the binding groove, which contributes to enhanced binding affinity with the human receptor that is characteristic of SARS-CoV-2 compared to SARS-CoV-1^74^. Despite that, deep mutational scanning suggests that the K417N replacement negatively impacts binding affinity to ACE2^75^. Also, Tegally et al.^71^ disclosed that the K417N mutation would abolish critical interactions with class 1 neutralizing antibodies and contribute to immune evasion at this site^71^. Glu484 is a critical residue within the RBM of RBD and interacts with the Lys31 residue of ACE2 and considered the site of most concern for viral mutations that impact binding and neutralization by polyclonal serum antibodies targeting the RBD^74,78^. Recently, the E484K replacement has been associated with escape from neutralizing antibodies.

### Virtual screening for drug repurposing

We performed virtual screening experiments with DockThor-VS for the e-Drug3D dataset at the reference pH (6.6 to 7.4) for all SARS-CoV-2 targets available at the platform so far (*e.g.,* PLpro, Mpro, RdRp, NendoU, Spike and N protein) using the wild type genomic variant. When the protein target has more than one conformation, we adopted an ensemble docking strategy to select the top-scored binding pose according to each drug’s predicted affinity (see Sect. 3.6 for details).

The virtual screening results of some drugs currently under clinical trials against COVID-19 are presented in Table 1. The compounds were selected apart of their main proposed mechanism of action and or the expected biological effect of the ongoing clinical trials.

**Table 1:** Virtual screening results of drugs ongoing clinical trials (accessed on 2020-09-03) for the drug targets available at DockThor-VS. Affinity predictions (kcal/mol) are given for the top-energy pose according to the ensemble docking strategy.

Name	# Studies^1^	PLpro	Mpro	RdRp	NendoU	N protein	Spike
Acetylcysteine	5	− 6.13	− 6.28	− 6.48	− 6.32	− 6.28	− **6.74**
Amodiaquine	1	− 7.57	− **8.23**	− 7.23	− 7.64	− 7.96	− 7.79
Atorvastatin	4	− 7.65	− **8.73**	− 7.37	− 6.92	− 7.97	− 8.71
Atovaquone	2	− 7.68	− 8.28	− 7.91	− **8.50**	− 8.33	− 8.12
Azithromycin	71	− 7.90	− 7.73	− 7.73	− 7.88	− 7.75	− **8.40**
Baricitinib	12	− 7.53	− 7.96	− 6.43	− **8.26**	− 7.98	− 7.61
Chloroquine	29	− 7.92	− 8.06	− 7.49	− 7.88	− **8.10**	− 7.65
Chlorpromazine	2	− 7.68	− **8.41**	− 6.74	− 7.71	− 7.45	− 7.61
Ciclesonide	4	− 8.04	− 8.68	− 7.42	− 8.45	− **8.92**	− 8.00
Cobicistat	2	− 8.36	− **9.46**	− 8.58	− 8.82	− 9.16	− 9.31
Daclatasvir	6	− 8.83	− 9.43	− 8.63	− 9.02	− **9.50**	− 9.02
Darunavir	3	− 8.07	− **8.45**	− 7.84	− 8.27	− 8.32	− 7.87
Deferoxamine	3	− 7.56	− 7.86	− 7.43	− 7.87	− **8.20**	− 7.81
Dexamethasone	18	− 7.28	− 7.83	− 6.90	− **8.01**	− 7.74	− 7.60
Disulfiram	1	− 7.23	− **7.80**	− 6.74	− 7.75	− 7.52	− 7.22
Eltrombopag	1	− 7.96	− 8.48	− 7.78	− 7.60	− **8.56**	− 8.40
Emtricitabine	3	− 6.47	− **7.03**	− 6.51	− 6.55	− 6.64	− 6.60
Fingolimod	1	− 7.96	− 7.46	− 6.83	− **8.10**	− 7.64	− 7.94
Hydroxychloroquine	185	− 7.76	− 7.83	− 7.42	− 7.66	− **8.10**	− 7.70
Ibuprofen	2	− 6.86	− **7.43**	− 6.70	− 7.26	− 7.19	− 6.89
Icatibant	1	− 6.95	− 7.58	− 8.97	− 7.50	− 8.34	− **9.04**
Imatinib	4	− 8.79	− **10.10**	− 7.69	− 8.78	− 8.66	− 8.61
Isotretinoin	5	− 7.04	− 7.20	− 6.93	− 7.03	− **8.03**	− 7.72
Ivermectin	36	− 8.37	− 9.26	− 8.62	− 8.94	− **9.44**	− 8.78
Ledipasvir	3	− 8.47	− **10.59**	− 9.16	− 9.25	− 9.36	− 9.66
Leflunomide	2	− 7.15	− **7.83**	− 7.13	− 7.79	− 7.42	− 7.17
Lopinavir	43	− 8.80	− 8.47	− 7.77	− **10.12**	− 9.06	− 8.76
Losartan	12	− 7.56	− 8.42	− 7.51	− **8.51**	− 8.39	− 8.03
Mefloquine	1	− **7.77**	− 7.46	− 7.03	− 7.49	− 7.17	− 7.47
Methylprednisolone	17	− 7.76	− 7.99	− 6.81	− **8.05**	− 7.74	− 7.68
Montelukast	1	− 8.38	− 9.08	− 8.65	− 8.52	− 9.02	− **9.28**
Niclosamide	3	− 7.44	− **8.04**	− 7.28	− 7.29	− 7.47	− 7.68
Nitazoxanide	19	− 7.41	− **7.77**	− 6.87	− 7.29	− 7.57	− 7.18
Oseltamivir	9	− 7.12	− 7.03	− 6.83	− 7.33	− 7.25	− **7.64**
Prazosin	2	− **8.84**	− 8.57	− 7.56	− 8.41	− 8.53	− 7.38
Ribavirin	7	− **6.91**	− 6.45	− 6.10	− 6.80	− 6.84	− 6.54
Ritonavir	48	− 8.69	− 8.70	− 7.63	− **9.00**	− 8.85	− 8.61
Ruxolitinib	19	− 8.16	− **8.41**	− 7.06	− 7.83	− 7.95	− 7.89
Sildenafil	2	− 8.26	− **8.64**	− 7.79	− 8.08	− 8.62	− 8.07
Sofosbuvir	7	− 7.92	− **8.49**	− 7.29	− 8.10	− 7.40	− 7.48
Telmisartan	9	− 8.55	− **9.38**	− 8.12	− 8.42	− 9.09	− 8.60
Tenofovir	1	− 6.86	− 6.89	− 6.86	− 6.65	− 6.73	− **6.90**
Thalidomide	2	− 7.15	− **7.40**	− 6.66	− 7.17	− 6.71	− 7.21
Tranexamic acid	4	− 6.34	− 6.59	− 6.15	− 6.18	− 6.54	− **7.12**

The top-scored drug for each target is underlined and the most promising target for each drug is highlighted in bold. The molecular weight (MW) and the number of rotatable bonds (RotB) of each compound are listed in Table S2.

^1^Number of clinical trials reported related to COVID-19 by Mapped Drug Intervention at the ClinicalTrials.gov (accessed on 2020-09-03, https://clinicaltrials.gov/ct2/covid_view/drugs).

The screening experiments primarily purpose in this work is to identify some drugs that could interact with one or more SARS-CoV-2 targets using a reverse docking protocol. However, it is important to state that a virtual screening campaign serves as a filter to identify the most probable molecules to affect the protein function. These molecules should be further validated/evaluated in experimental assays and clinical trials to confirm their biological/therapeutic efficacy. Moreover, the top-scored molecules' binding mode could be visually inspected and subjected to more sophisticated simulation techniques before going to the more expensive and time-consuming experimental validation step. Thus, the docking results present in this work must not be used for guiding clinical practice.

Another relevant point is that some drugs currently ongoing clinical trials listed on Table 1 do not have evidence related to antiviral properties, but are claimed to be important in treating, for example, the cytokine storm such as dexamethasone. However, it is also possible that some drugs with known mechanisms of action, related to the interaction with host proteins or even with the virus entry pathway, can also inhibit one or more targets of the pathogen, thus exhibiting multiple mechanisms for the antiviral effect.

Considering all targets and all drugs evaluated, we found that the majority of the predicted binding affinities are moderate in the range of high to low micromolar affinity units (scores higher than -6.8 kcal/mol correspond to binding affinity values higher than ~ 10 µM, whereas scores lower than -8.2 kcal/mol corresponds to submicromolar affinities). This result is expected since many drugs under clinical trials exhibited no activity or only modest inhibitory effects against SARS-CoV-2 in some experimental studies reported in the literature^79–81^.

However, we found some interesting results that deserve to be highlighted. We identified ledipasvir, imatinib, lopinavir and daclatasvir as the most promising drugs under clinical trials. They all show a good multi-target profile and exhibit some predicted binding affinities at a low nanomolar concentration (Table 1). Ivermectin, montelukast, posaconazole, ritonavir and telmisartan also show an interesting multi-target profile having predicted binding affinities below sub-micromolar concentration for at least five targets.

Ledipasvir is an antiviral to treat chronic Hepatitis C targeting the non-structural protein 5A from the HCV (NS5A) and was predicted as the most potent Mpro inhibitor (score = -10.59 kcal/mol) among the drugs currently ongoing clinical trials against COVID-19. Also, ledipasvir was predicted as the most potent drug under clinical trials against Spike and RdRp (scores of -9.66 kcal/mol and -9.16 kcal/mol, respectively). Daclatasvir was the top-ranked drug against the N protein (score = -9.50 kcal/mol). Daclatasvir is also an inhibitor of NS5A from HCV and might also be a promising Nsp5 inhibitor, with a predicted binding score of -9.43 kcal/mol. Lopinavir is another antiviral drug inhibitor of the HIV-1 protease administered in combination with other antiretrovirals in the treatment of AIDS. Herein, it was predicted as the most potent drug against Nsp15 in the virtual screening experiments.

The anticancer imatinib is an Abl kinase inhibitor with an immune-modulatory effect that was reported to exhibit anti-SARS-CoV action by blocking the viral envelope fusion with the cell membrane^82^, being an interesting drug that might have a dual-purpose effect. According to the virtual screening results, it also potentially inhibits the SARS-CoV-2 Mpro target with a docking score of -10.10 kcal/mol, interacting at the binding site with key residues such as His163 and Met49. Imatinib was previously reported to inhibit SARS-CoV and MERS infections in cell culture assays^83^. However, a recent study reported that imatinib had no effects on SARS-CoV-2 infection and replication in a standard viral replication assay^84^.

Other examples of drugs proposed to be involved in the symptomatic treatment but obtained good scores against at least one SARS-CoV-2 target are atorvastatin and prazosin.

Atorvastatin is an oral antilipemic agent that inhibits the HMG-CoA reductase and also possess anti-inflammatory and immune-modulatory effects, being the statins suggested as effective against viral infections^85–87^ and associated with reduced disease severity of COVID-19 infection^88^. According to our screening protocol, atorvastatin achieved moderate affinities against both Mpro and Spike proteins (predicted affinities of − 8.73 kcal/mol and − 8.71 kcal/mol, respectively). Together with the other reported beneficial effects, if its anti-SARS-CoV-2 action is confirmed by experimental methods, atorvastatin could be a very promising drug to be repurposed against COVID-19 due to its pleiotropic effect and low cost.

Prazosin is an alpha-1 antagonist used to treat hypertension that is currently under clinical trials to evaluate its efficacy and safety in preventing the COVID-19 cytokine storm. In our virtual screening experiments, it was predicted as the best Nsp3 drug among those currently under clinical trials with a moderate docking score of − 8.84 kcal/mol.

Ivermectin is an antiparasitic drug with reported in vitro antiviral effects against SARS-CoV-2, with a reduction of 99.98% in viral load in Vero/hSLAM cells after 48 h of treatment^89^, with the antiviral effect hypothesized to be due to the inhibition of nuclear import of viral proteins targeting the host importin α/β1 heterodimer^90^. In our screening experiments, Ivermectin achieved good scores against all SARS-CoV-2 targets evaluated in our screening experiments, mainly N protein (− 9.44 kcal/mol) and Mpro (− 9.26 kcal/mol), suggesting that it could exhibit an on-target antiviral effect inhibiting SARS-CoV-2 proteins. Indeed, other mechanisms of action for ivermectin are reported in the literature, including the interaction with the NS3 helicase from dengue virus^91^.

The moderate (> − 8.2 kcal/mol) to low (> − 6.8 kcal/mol) binding affinities predicted for some drugs with antiviral activities found experimentally suggest that their primary mechanism of action might not be due to the interaction with SARS-CoV-2 targets, at least for the proteins evaluated in our experiments. Examples of off-target mechanisms to achieve antiviral effects include the inhibition of clathrin-mediated endocytosis (*e.g.,* the antipsychotic drug chlorpromazine^92–95^) and the lysosomotropic effect (*e.g.,* the antimalarial drugs chloroquine and hydroxychloroquine^96^) and on-target mechanisms interfering in the function of host proteins such as ACE-2 and TMPRSS2 (*e.g.,* the serine protease inhibitor camostat-mesylate^97^). Until now, there is no evidence that such exemplified drugs inhibit SARS-CoV-2 protein targets.

We also evaluated the top-20 drug candidates for repurposing that are not ongoing clinical trials for each SARS-CoV-2 target (Table S3). One of the most interesting results is associated with the antiviral drug elbasvir, which was predicted to interact with both Mpro and N protein at docking scores lower than − 10 kcal/mol, suggesting a multi-target effect of this drug. In addition to Mpro and N protein, elbasvir also was predicted to be within the top-20 drugs for other SARS-CoV-2 targets, *i.e.,* Spike (− 9.79 kcal/mol), NendoU (score = − 9.61 kcal/mol) and RdRp (score = − 9.21 kcal/mol).

None of the top-ranked drugs to target Nsp3 was predicted with binding affinities lower than − 10 kcal/mol. However, we can highlight bazedoxifene (score = − 9.65 kcal/mol) and menaquinone (score = − 9.50 kcal/mol) as interesting findings. Bazedoxifene is a selective estrogen receptor modulator used to prevent postmenopausal osteoporosis, and strong antiviral effects have been reported for SARS-CoV-2^79,98^. Due to its inhibitory effect on IL-6 signalling, bazedoxifene has been proposed as a promising drug to prevent the cytokine storm, ARDS and mortality in severe COVID-19 patients^99–101^. Menaquinone (Vitamin K2) is one of the three types of Vitamin K, and a recent preliminary study suggested that patients with Vitamin K deficiency were related to poor prognostic^102^. However, there is no yet evidence if menaquinone administration helps to treat or prevent COVID-19 infection.

**Figure 8:**
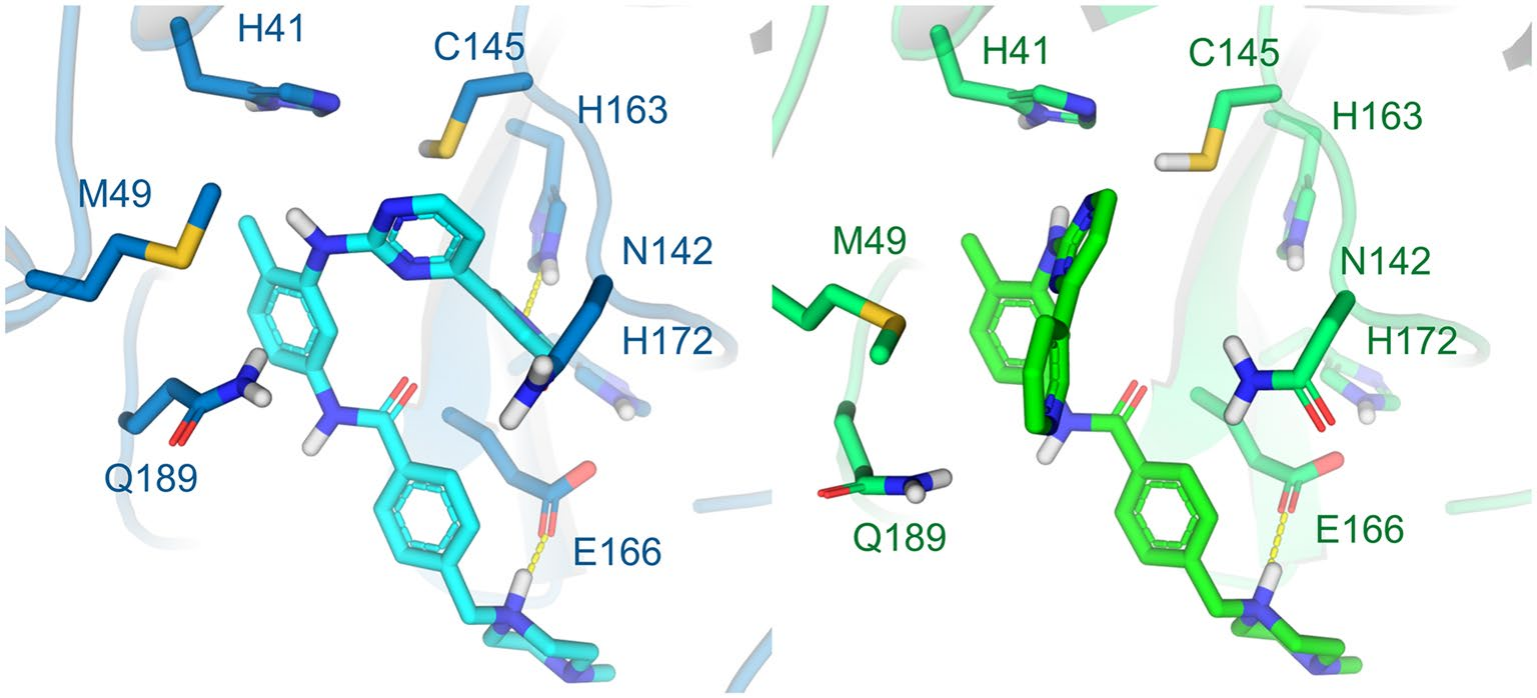
Predicted binding modes of imatinib against the Mpro conformations 6LU7 (left, score = − 10.09 kcal/mol, imatinib represented as cyan sticks) and 6W63 (right, score = − 8.80 kcal/mol, imatinib represented as green sticks). Hydrogen bonds are represented as yellow dashed lines.

The Mpro screening predicted six antiviral drugs within the top-20 best-affinity compounds, where two of them (*i.e.,* ledipasvir and velpatasvir) are currently ongoing clinical trials. The anti-HCV drugs elbasvir, pibrentasvir, velpatasvir and ombitasvir are still not being evaluated in clinical trials but could be promising drugs for repurposing to fight against COVID-19. The comparison between the experimental structures of Mpro suggests significant conformational changes of amino acid side chains within the binding site, especially the residues Met49, Asn142, Met165 and Gln189. Imatinib is an example compound that has both affinity and binding pose predictions affected by the receptor conformation, interacting better in the 6LU7 conformation (score = − 10.09 kcal/mol, ranked 5th best compound) than in 6W63 (score = − 8.80 kcal/mol, ranked at the 100th position). In the 6LU7 conformation, the pyridine group of imatinib can interact deeply in the Mpro binding site, making a hydrogen bond with His163. In contrast, in the 6W63 conformation, this pyridine moiety is exposed to the solvent (Fig. 8). Other examples that the virtual screening ranking was also strongly affected by the receptor conformation are posaconazole against PLpro (ranked 7th in 6W9C and 1054th in 6WX4) and elbasvir against the NMR-derived conformations (ranked 1st in the state-12 and ranked 10th in the state 10). These results show the importance of using various, carefully selected, conformations of a protein target in virtual screening experiments. Using only one particular receptor conformation could generate false-negative results: (i) by discarding promising ligands due to a lousy ranking position; (ii) by inducing an incorrect ligand binding mode that could harm further fully flexible interaction analysis of the protein–ligand complexes through molecular dynamics simulations currently used by many groups^18,20^.

The best-scored drug against RdRp was the anticancer dactinomycin (score = − 9.88 kcal/mol), a macrocyclic drug that binds to DNA inhibiting the synthesis of RNA. Associated with the screening result, its mechanism of action might suggest that dactinomycin can interact at the RNA binding site of RdRp, which is currently available in the DockThor-VS platform in the free form (*i.e.,* without primer and metal ions observed in the 7BV2 structure). Ribavirin, a known RdRp inhibitor, was predicted with a weak affinity score = − 6.10 kcal/mol) probably due to the concerted mechanism of action with the primer and metal ions.

The antiviral lopinavir was the only drug that achieved a docking score lower than − 10 kcal/mol against NendoU. Despite this, we highlight lomitapide (score = − 9.96 kcal/mol), a drug widely used to treat familial hypercholesterolemia that was recently found to exhibit anti-SARS-CoV-2 activity on traditional CPE-based antiviral assay in Vero E6^103^. Additionally, lomitapide was also predicted to inhibit Spike with a docking score of − 9.26 kcal/mol, suggesting a multi-target potential.

Elbasvir and fidaxomicin were predicted to interact with N protein with docking scores of -10.21 kcal/mol and 10.00 kcal/mol, respectively. As mentioned before, elbasvir is an antiviral drug predicted to interact favorably with multiple SARS-CoV-2 targets. Fidaxomicin is a macrocyclic lactone antibiotic drug with activity against *C. difficile* targeting the bacterial RNA synthase^104^. If the fidaxomicin's antiviral effect against SARS-CoV-2 were confirmed, it could be promising for drug repurposing. It has almost no impact on the normal gut microbiome and is certified for pediatric use in patients over six months^105^.

Ombitasvir was the top-ranked compound against Spike and the only compound achieving a docking score better than -10 kcal/mol. It is an antiviral drug targeting NS5A in treating chronic cases of Hepatitis C in combination with other antiviral compounds. To date, no experimental studies have reported anti-SARS-CoV-2 effects. However, it might be a promising drug candidate for repurposing due to similar action mechanisms to other NS5A inhibitors already registered to have anti-SARS-CoV-2 activity. Furthermore, ombitasvir were predicted to interact with Mpro and NendoU at nanomolar concentration, suggesting that it can also exhibit a multi-target effect on SARS-CoV-2.

### Impact of non-synonymous variations in virtual screening

**Figure 9:**
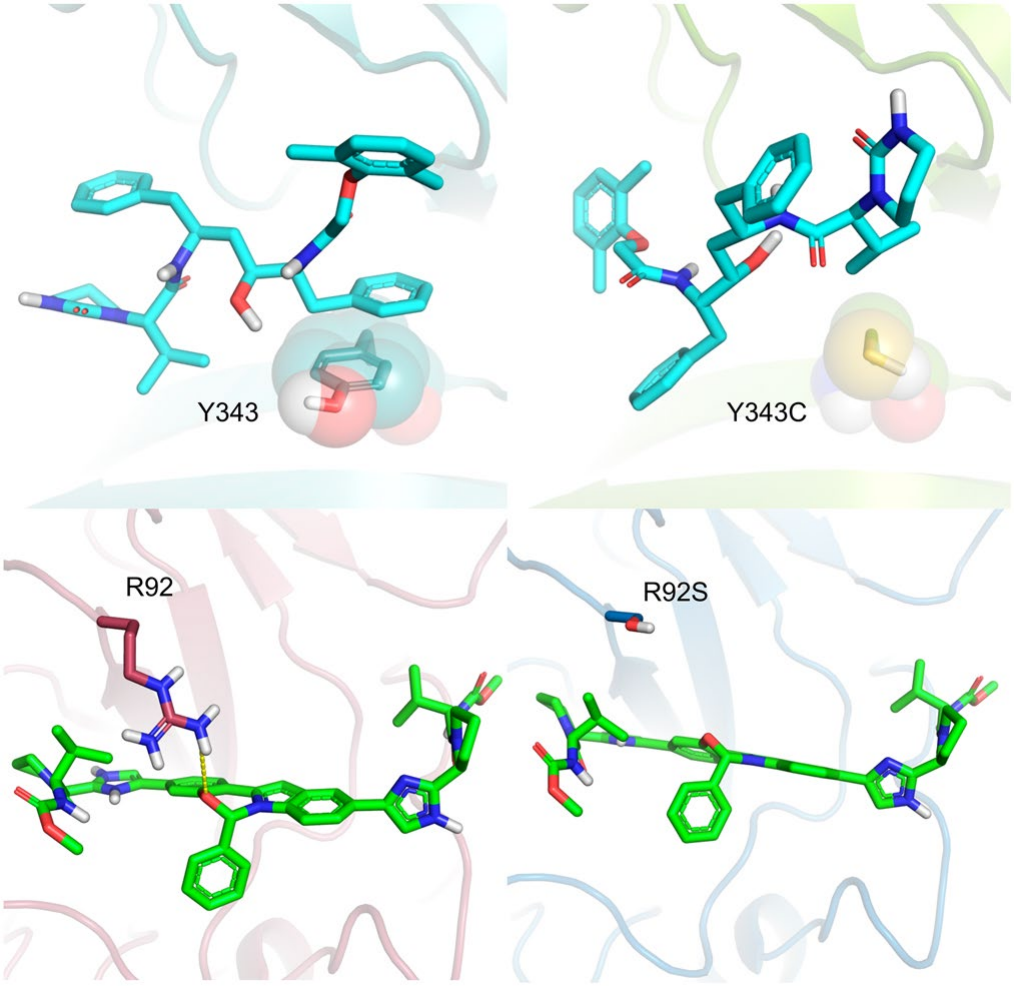
(**A**) Docking result of lopinavir against the wild type (left) and Y343C mutant (right) of NendoU. (**B**) Docking result of elbasvir against the wild type (left) and Y343C mutant (right) of N protein (state 12).

One of the possible impacts of non-synonymous variations is on the conference of resistance to some drugs by changing the interactions profile performed between the compound and key amino acid residues at the binding site. NendoU-lopinavir is an interesting example to evaluate the influence of the non-synonymous mutation Y343C in the predicted binding mode and affinity for this complex (Fig. 9A). Lopinavir was predicted to interact with wild type NendoU deeply in the binding site with a docking score of -10.12 kcal/mol, characterized by interactions with crucial residues such as π-stacking between its phenyl ring and the Try343 side chain. As expected, the Y343C variation led to the loss of this interaction and a worse predicted affinity (− 9.33 kcal/mol). Lopinavir interacts superficially in the binding cavity without reaching key residues such as Tyr343 and Ser294, whereas still making favorable interactions with the receptor. N protein-elbasvir is another interesting example, where the R92S variation weakened the predicted binding affinity (score_WildType_ = − 10.21 kcal/mol versus score_R92S_ = − 9.68 kcal/mol) led to the loss of the hydrogen-bonded formed between the oxygen-containing heterocycle from elbasvir and the Arg92 side chain in the wild type N-protein, however without significantly changing the predicted binding mode (Fig. 9B).

We also performed virtual screening experiments for the Spike variants explored in our work (i.e., K417N, N439K, F456L, G476S, V483A, E484K, N501Y, and N501Y + K417N + E484K), totalizing 24 screening due to the ensemble docking protocol using the three distinct Spike conformations (Table 2). The mutants lead to specific effects on the top-ranked drugs, in general without significant differences in the predicted affinities. Ombitasvir, the top-scored drug against the wild type Spike, was negatively impacted by N439K, achieving a drop in the predicted affinity of almost 1 kcal/mol. Still, some compounds were predicted to be even more potent against some variants when compared to the wild type protein, such as elbasvir, mivacurium, cetrorelix and abarelix. According to the affinity, the drugs' ranking order also changed compared to the wild type structure, but in general, the compounds remain the same. Examples of drugs (Table S4) predicted as the top-1 drug against some variant but not present in the wild type Spike top-20 list are leuprolide (K417N), cisatracurium (G476S) and triptorelin (E484K). However, protein dynamic effects caused by the mutations, not taken into account by the docking protocol, can significantly affect these results.

**Table 2:** Affinity prediction of the top-20 drug candidates for repurposing against Spike wild type and for each variant evaluated (*i.e.*, K417N, N439K, F456L, G476S, V483A, E484K, N501Y, and Multiple = N501Y + K417N + E484K).

Name	Wild Type	K417N	N439K	F456L	G476S	V483A	E484K	N501Y	Multiple
OMBITASVIR	− 10.08	− 9.95	− 9.00	− 9.77	− 9.70	− 9.83	− 9.91	− 9.77	− 9.80
ELBASVIR	− 9.79	− 10.37	− 10.48	− 10.21	− 10.43	− 9.89	− 9.84	− 10.46	− 9.97
NAFARELIN	− 9.78	− 10.00	− 10.12	− 9.24	− 9.98	− 10.04	− 10.17	− 9.43	− 10.28
BETA-CAROTENE	− 9.72	− 9.62	− 9.15	− 9.21	− 9.33	− 9.36	− 9.64	− 9.41	− 9.59
PIBRENTASVIR	− 9.70	− 9.32	− 10.08	− 10.01	− 9.81	− 9.77	− 9.51	− 9.19	− 9.94
LEDIPASVIR	− 9.66	− 10.27	− 10.07	− 10.21	− 10.41	− 10.24	− 10.16	− 10.53	− 9.93
VELPATASVIR	− 9.56	− 9.40	− 9.55	− 9.59	− 9.10	− 9.57	− 9.44	− 9.38	− 9.55
GOSERELIN	− 9.48	− 8.85	− 9.18	− 9.41	− 9.99	− 9.64	− 10.05	− 8.93	− 9.11
PALIPERIDONE-PALMITATE	− 9.39	− 9.55	− 9.24	− 9.21	− 9.15	− 9.09	− 9.68	− 9.26	− 8.94
MIVACURIUM	− 9.39	− 9.75	− 10.44	− 10.58	− 9.67	− 9.72	− 9.99	− 9.53	− 9.86
PLICAMYCIN	− 9.38	− 9.27	− 9.47	− 9.60	− 9.22	− 9.11	− 9.67	− 9.37	− 9.45
CETRORELIX	− 9.34	− 10.61	− 9.79	− 10.47	− 9.37	− 10.08	− 9.81	− 10.13	− 10.21
COBICISTAT	− 9.31	− 9.61	− 9.00	− 9.27	− 9.41	− 9.11	− 9.34	− 9.65	− 9.43
ABARELIX	− 9.30	− 9.42	− 10.95	− 9.70	− 10.13	− 10.81	− 9.75	− 10.62	− 9.77
MONTELUKAST	− 9.28	− 9.18	− 8.79	− 8.87	− 9.12	− 8.93	− 9.03	− 9.02	− 9.07

^1^Number of clinical trials reported related to COVID-19 by Mapped Drug Intervention at the ClinicalTrials.gov (accessed on 2020-09-03, https://clinicaltrials.gov/ct2/covid_view/drugs).

In this context, the availability of both wild type and selected non-synonymous variations mainly present in the binding sites targeted by small molecules could be handy for the drug design of more potent and effective compounds to fight against relevant virus variants.

## Methods

### Analyses of non-synonymous variations

The SARS-CoV-2 genomic sequences were obtained from the GISAID database (www.gisaid.org). The identification of a non-synonymous variation (NSV) in the selected targets was performed according to the CoV-GLUE database^65^ (http://cov-glue.cvr.gla.ac.uk, accessed on July 7, 2020). We annotated for each residue containing NSV the physicochemical properties of the related amino acids in both the Wuhan-Hu-1 reference sequence (NCBI NC_045512.2) and the genome sequence recovered from GISAID. The prediction of the biological impact of NSV (deleterious or neutral) was estimated from homology data combined with BLOSUM62 substitution matrices provided by the PROVEAN algorithm (http://provean.jcvi.org/index.php). For features annotation in the amino acid sequence, we used several databases, such as PDB and InterPro (https://www.ebi.ac.uk/InterPro/search/sequence) and UniProt-covid19 (https://covid-19.uniprot.org). Also, we sought carefully and thoroughly in the literature for mutagenesis experiments with evidence of alteration in the protein molecular function or viral fitness in CoVs involving the focused residue.

### Preparation of the protein structures

We provided the DockThor-VS users with the structures of some SARS-CoV-2 potential therapeutic targets for the design of new drugs and vaccines. For this purpose, we initially select the non-structural proteins Nsp3, Nsp5 (PLpro domain), Nsp12 (RdRp) and Nsp15 (endoribonuclease), as well as the structural proteins Spike and nucleocapsid protein (N protein). For the N protein, we clustered 31 conformations with Glu174 present in an opened conformation out of a total of 40 states present in the NMR-derived structure (PDB code 6YI3^51^) to select a small subset representative of the protein flexibility. The aliphatic carbon atoms from the Glu174 side chain are part of the phosphate-binding site, and the closed conformation might lead to steric clashes with potential inhibitors in the binding site. For this purpose, we clustered the opened states (31 out of 40 states) using the Conformer Cluster tool in Maestro (Maestro, Schrödinger, LLC, New York, NY, 2020) according to the position of the residues Arg102 and Tyr109 using the weighted centroid as the linkage method. Finally, the nearest to the centroid structure per cluster was selected as the representative conformation of each group available at DockThor-VS.

In this work, we prepared the protein structures using the Protein Preparation Wizard from Maestro (Schrödinger Release 2020–2: Maestro, Schrödinger, LLC, New York, NY, 2020)^106^. Protonation assignment and hydrogen-bond optimization were performed using ProtAssign and PROPKA^107^ at the reported experimental pH and considering the presence of the bound ligand when available. Metal ions were considered cofactors when necessary, whereas water molecules and ligands originally present in the experimental structures were removed. The protonation/tautomeric states of the binding-site residues and the bound ligand were further visually inspected, and appropriate corrections were made guided by the reaction mechanism of the protein target described in the literature.

In silico point mutation for each of the variations was done using Modeller^108^. First, an extended model is created, using standard topologies, for the mutated sequence. After that, all possible atomic coordinates are transferred from the wild model to the new mutated model. The missing coordinates are rebuilt using the standard topologies. The new sidechain atoms are randomly displaced by at most 4.0 Å and then optimised by two gradient descent runs. The mutated sidechain was further refined by a short round of Modeller's molecular dynamics. Cognate non-covalent ligands were maintained throughout the protocol when necessary.

### Preparation of the datasets with known drugs

#### e-Drug3D

The dataset containing the FDA-approved drugs and active metabolites was constructed from the e-Drug3D dataset, a dataset updated annually and freely available for the scientific community at https://chemoinfo.ipmc.cnrs.fr/MOLDB/index.php^109,110^. The e-Drug3D is an essential dataset for many drug design efforts such as drug repurposing and was carefully constructed with high-quality and curated structures of FDA-approved drugs and active metabolites. In the June 2019 updated version, there are 1930 structures of approved drugs and active metabolites with molecular weight less than 2000. The list of the grouped active metabolites with the respective approved drug can be accessed at https://chemoinfo.ipmc.cnrs.fr/MOLDB/metabolite_groups.html. Small macrocyclic drugs (backbone with less than 20 heavy atoms) are provided to the users in the original conformation present in the e-Drug3D dataset. The ensemble of conformations of small macrocyclic drugs from e-Drug3D is supplied in a separated dataset.

#### Drugs under clinical trials (COVID-19 repurposing dataset)

The dataset of drugs under clinical trials was collected from published articles^14,111–114^ and approved drugs listed on the DrugBank database in the "Clinical Trial Summary by Drug" section. Small macrocyclic drugs (backbone with less than 20 heavy atoms) are provided to the users in the original conformation present in the e-Drug3D dataset. The ensemble of conformations of small macrocyclic drugs from e-Drug3D is supplied in a separated dataset.

#### Macrocycles dataset

A separate dataset for e-Drug3D macrocycle drugs (with backbone size smaller than 20 heavy atoms) is also provided to the users. This dataset contains distinct macrocycles conformations, representing to some extent the molecular macrocycle flexibility. The macrocycle conformational sampling was performed with the Prime macrocycle conformational sampling tool from the Schrödinger 2020–2 suite to generate up to 10 conformers for each input structure.

#### Protonation and tautomeric states

The most probable protonation and tautomeric states of the compounds in the datasets were predicted with Epik from the Schrödinger 2020–2 suite^106,115^. The Epik predictions were based on the pKa estimation of the ionizable groups on aqueous solvent, considering probable metal-binding states. A maximum of 16 output structures for each input structure was allowed at a reference pH (6.6 to 7.4). Protonation states at low (4.0 to 6.5) and high (7.5 to 10.0) pH ranges to cover diverse situations observed for many therapeutic targets will be provided soon.

### The DockThor-VS platform

The DockThor-VS web server is based on a suite of programs mainly developed by our research group GMMSB. The docking engine is the DockThor program^25,26^, a non-covalent docking program that utilizes a topology file for the ligand and cofactors (*.top*) and a specific input file for the protein (*.in*) containing the atom types and partial charges from the MMFF94S49 force field. The .top file of the ligand is generated by the *MMFFLigand* program, which utilises the facilities of the OpenBabel chemical toolbox^116^ for deriving partial charges and atom types with the MMFF94S force field^117^, identification of the rotatable bonds and the terminal hydroxyl groups, and calculating the properties necessary for computing the intramolecular interactions. In the *MMFFLigand*, all hydrogen atoms are considered explicitly. The *PdbThorBox* program is used to set the protein atom types, the partial charges from the MMFF94S force field considering the nonpolar atoms implicitly, and reconstruct missing residue side-chain atoms. Thus, in the DockThor-VS platform, both protein, cofactor and ligands are treated with the same force field in the docking experiment. All the molecular force field parameterisations are performed automatically by the programs cited without the users’ need for intervention. However, some preparation steps of the input molecules can be done interactively in the web server, such as changing the protonation state of some amino acid residues, adding hydrogen atoms and freezing rotatable bonds of the ligand (only available for the upload of a single ligand).

The search space is represented as a grid box where the potentials are stored at the grid points, significantly reducing the computational cost. The user can interactively set the grid box’s configuration in the web server through the parameters: center of coordinates, size of the grid and discretization (*i.e.,* the spacing between the grid points). The initial population is randomly generated within the grid box using random values for the rotational, translational, and conformational degrees of freedom of the ligand. For each SARS-CoV-2 therapeutic target, DockThor-VS provides a recommended set of parameters for the grid box (*i.e.*, center and grid sizes) that the user can use or modify according to the objectives of his docking experiment (Table S5).

The DockThor docking program was specially developed to deal with highly flexible ligands, and it is very suitable to dock highly flexible peptides^26^. The program uses a phenotypic crowding-based multiple solution steady-state genetic algorithm as the search method^25^. In this strategy, the parental replacement method follows the Dynamic Modified Restricted Tournament Selection (DMRTS), which provides a better exploration of the energy hypersurface and allows identifying multiple minima solutions in a single run, preserving the population diversity of the generated structures. The default parameters of this algorithm (named *Standard*) are set in the web server as follows: (i) 24 docking runs, (ii) 1.000.000 evaluations per docking run, (iii) population of 750 individuals, (iv) maximum of 20 cluster leaders on each docking run. For virtual screening experiments, we also provide an alternative set of parameters to accelerate the docking experiment without significantly losing accuracy (named *Virtual Screening*): (i) 12 docking runs, (ii) 500.000 evaluations per docking run, (iii) population of 750 individuals, (iv) maximum of 20 cluster leaders on each docking run. The docking experiments are performed on CPU nodes of the SDumont supercomputer, each one containing two processors Intel Xeon E5-2695v2 Ivy Bridge (12c @2,4 GHz) and 64 Gb of RAM memory. We validated the docking experiments through the redocking of the non-covalent ligands present in the complexes 6W63 (Mpro) and 6WXC (Nendo-U) using the *standard* configuration, successfully predicting the co-crystallized conformation of each complex (Table S5). The experimentally observed interactions between the uracil ring from tipiracil and Nendo-U binding site were correctly predicted for the top-energy solution of tipiracil. In contrast, the iminopyrrolidin was predicted on an inverted conformation to optimize the interactions with the protein. In the crystallographic structure, this moiety is exposed to the solvent and has insufficient electronic density data.

The scoring function used to score the docked poses of the same ligand is based on the sum of the following terms from the MMFF94S force field and is named “Total Energy (Etotal)”: (i) intermolecular interaction energy calculated as the sum of the van der Waals (buffering constant δ = 0.35) and electrostatic potentials between the protein–ligand atom pairs, (ii) intramolecular interaction energy calculated as the sum of the van der Waals and electrostatic potentials between the 1–4 atom pairs, and (iii) torsional term of the ligand. All docking poses generated during the docking step are then clustered by the in*-*house tool *DTStatistics.* The top energy-poses of each cluster are selected as representatives and made available to the users. In the results analysis section, users can choose the clustering criteria and the maximum number of cluster representatives available on the web server. The affinity prediction and ranking of distinct ligands are performed with the linear model and untailored for specific protein classes, *DockTScore*_*GenLin*_ scoring function. The DockTScore is a set of empirical scoring functions recently developed by our research group^118^. These scoring functions take into account the following important terms for protein–ligand binding: (i) intermolecular interactions terms; (ii) a torsional entropy term that penalizes the "frozen" rotatable bonds due to binding, (iii) a protein–ligand lipophilic interaction term, (iv) a polar solvation term which accounts for the loss of polar interactions of the charged groups of both protein and ligand after binding and (v) a favorable nonpolar solvation term that is proportional only to the solvent-accessible surface. The scoring function was trained and tested in a large set (> 2900) of high-quality 3D structures associated with diverse physicochemical profiles (including an extensive range of binding affinities, MWs and number of rotatable bonds) and relevant therapeutic targets for drug design. In addition to the current scoring function, shortly we will also provide the affinity prediction using models developed for specific target classes such as proteases and protein–protein interactions (PPIs) and trained with sophisticated machine-learning algorithms.

The DockThor program is freely available as a web server at www.dockthor.lncc.br, which provides to the user the main steps for protein and ligand preparation with PdbThorBox and MMFFLigand, and the analyses of the results using DTStatistics. The visualization of protein, cofactors and compounds, the grid location superposed with the protein and the docking results are generated with NGL, a WebGL-based library for molecular visualization^119^. Guest users are allowed to submit VS experiments with up to 200 compounds, whereas registered users with approved projects can submit up to 5,000 compounds per job. All users have access to the COVID-19 repurposing and Macrocycle datasets, but only registered users can access the complete e-Drug3D repurposing dataset. The web server utilises the computational facilities of the Brazilian high-performance platform (SINAPAD, https://www.lncc.br/sinapad/) and the supercomputer SDumont (https://sdumont.lncc.br/).

## Conclusions

In the present work, we have described our current efforts to improve and apply a virtual screening approach at the DockThor-VS platform to repurpose known drugs against six selected proteins of SARS-CoV-2. A relevant aspect of this study is the availability on the platform of both the target models related to the reference sequence Wuhan-Hu-1 (wild type) and several non-synonymous variants selected from the epidemiological scenario.

In this work, we also observe the importance of using various, carefully selected, conformations of the protein target in virtual screening experiments to decrease the possibility of false-negative results. The use of only one particular receptor conformation can generate a bad ranking position for promising ligands or predict an incorrect ligand binding mode that could harm further fully flexible interaction analysis of the protein–ligand complexes.

For some non-synonymous variations, we also analyzed the possible impacts in the conference of resistance to some drugs promoted by changing the ligand–protein interaction profile. The availability of both wild type and selected non-synonymous variations could be beneficial for the drug design of more effective compounds having a broader spectrum against SARS-CoV-2 genomic variants.

Currently, the DockThor-VS platform provides curated 3D structures for wild type and selected mutations for papain-like (PLpro domain), Mpro, RdRp, NendoU, N protein and Spike. Given the increasing amount of 3D data available at the Protein Data Bank (PDB), we are continually updating the targets dataset, and other structures of new targets will be available soon. DockThor-VS will also be continuously updated with new docking methodologies developed at our research group, including new strategies for protein flexibility and binding affinity prediction.

We believe that the DockThor-VS web server is an excellent alternative for the development of drug repositioning research against COVID-19, not only for constructing drug libraries and therapeutic target structures available through the web server, but also for allowing studies involving new targets structures provided by the users and virtual screening experiments involving in-house ligand libraries. Moreover, the results obtained using the docking methodologies provided by DockThor-VS can be used in conjunction with the results of other docking programs in methodological approaches known in the literature as consensus scoring^120^ and consensus ranking^16^. The DockThor-VS web server is freely available at www.dockthor.lncc.br and utilizes the computational facilities of the Brazilian high-performance platform and the supercomputer SDumont (https://sdumont.lncc.br/).

## Supplementary Information

Supplementary Information

## Data Availability

The DockThor-VS platform is freely available for the scientific community at www.dockthor.lncc.br supporting virtual screening experiments with up to 200 compounds for guest users and 5,000 compounds for registered projects. The three-dimensional structures of the SARS-CoV-2 targets PLpro, Mpro, RdRp, NendoU, Spike and N protein, both wild types and molecular models of the selected non-synonymous variations, will be freely available at the DockThor-VS web server as *Protein Data Bank* format (.pdb).
