# Supplementary material for: Drug design and repurposing with DockThor-VS web server focusing on SARS-CoV-2 therapeutic targets and their non-synonym variants
Source: Sci Rep. 2021 Mar 10;11:5543. doi: 10.1038/s41598-021-84700-0 (PMC7946942; doi:10.1038/s41598-021-84700-0)
Supplement: Supplementary file 1 — Supplementary Information [file 41598_2021_84700_MOESM1_ESM.pdf]

Supplementary information

Drug Design and Repurposing with DockThor-VS Web Server focusing on SARS-CoV-2 Therapeutic Targets and their Non-Synonym Variants

Isabella A. Guedes [1], Leon S. C. Costa [1], Karina B. dos Santos [1], Ana L. M. Karl [1], Gregório K. Rocha [2], Iury M. Teixeira [1], Marcelo M. Galheigo [1], Vivian Medeiros [1], Eduardo Krempser [3], Fábio L. Custódio [1], Helio J. C. Barbosa [1], Marisa F. Nicolás [4,*] and Laurent E. Dardenne [1,*]

[1] Grupo de Modelagem Molecular em Sistemas Biológicos (GMMSB), National Laboratory for Scientific Computing - LNCC, Petrópolis - RJ, Brazil;
[2] Instituto Federal Fluminense - IFF, Macaé - RJ, Brazil;
[3] Fundação Oswaldo Cruz - Fiocruz, Rio de Janeiro - RJ, Brazil;
[4] Laboratório de Bioinformática (Labinfo), National Laboratory for Scientific Computing - LNCC, Petrópolis - RJ, Brazil;
* dardenne@lncc.br (L.E.D.), marisa@lncc.br (M.F.N.)

Table S1. SARS-CoV-2 target proteins and the non-synonymous variations (NSVs) selected in this study.

ORF	Description	NSVs[1]	Mutations Features[2,3]
Nsp3	Papain-like protease, PLpro	P993S (P248S)	Amino-acid substitution (hydrophobic to hydrophilic change) with neutral functional effect, which was identified in four samples belonging to either sub-lineages B, B.1, B.1.5, B.1.177.19, B.6 or B.18. The residue falls on the Peptidase C16 domain (UniProt P0DTD1).
		Y1009H (Y264H)	Amino-acid substitution (hydrophobic to moderate and neutral to positive changes) with neutral functional effect, which was identified in several samples belonging to either sub-lineages B, B.1, B.1.1, B.1.1.1 B.1.23, B.1.33, B.1.36, B.1.177, B.2, B.2.1, B.2.4, B.2.6, B.4 or B.6. The residue falls on the Peptidase C16 domain (UniProt P0DTD1).
		T1046M (T301M)	Amino-acid substitution (hydrophilic to moderate change) with neutral functional effect, which was identified in several samples belonging to either sub-lineages B. B.1, B.1.160, B.1.177 or B2. The residue falls on the Peptidase C16 domain (UniProt P0DTD1).
Nsp5	Main protease, Mpro	M165I	Amino-acid substitution (moderate to hydrophobic change) with neutral functional effect, which was identified in a sample belonging to either sub-lineage B, B.1.1, B.1.36, B.1.1.77, B.1.1.277. The residue falls on a beta-sheet and is

			directly part of the ligand-binding site, with the side chains oriented towards the ligand.
Nsp12	RNA-dependent RNA polymerase, RdRp	G683V	Amino-acid substitution (no amino-acid properties change) with deleterious functional effect, which was identified in a sample belonging to sub-lineage B.1. The residue falls on the active site of the RdRp domain (motif B), which is formed by the conserved polymerase motifs A-G in the palm domain and configured like other RNA polymerases.
Nsp15	Endoribonuclease NendoU	S293A (S294A)	Amino-acid substitution (hydrophilic to hydrophobic change) with neutral functional effect, which was identified in several samples belonging to either sub-lineages B, B.1.p11, B.1.1, B.1.5, B.1.5.6, B.1.36, B.1.160, B.1.177, or B.2.1. The residue falls on the ligand interaction binding site. Ser293 accounts as the key residue for enzyme discrimination between uracil to cytosine or adenine to guanine bases.
		S293T (S294T)	Amino-acid substitution (no amino-acid properties change) with neutral functional effect, which was identified in several samples belonging to either sub-lineages B.1.5, B.1.14, B.1.36, B.6 or B.14. The residue falls on the ligand interaction binding site. Ser293 accounts as the key residue for enzyme discrimination between uracil to cytosine or adenine to guanine bases.
		Y342C (Y343C)	Amino-acid substitution (hydrophobic to moderate and aromatic to non-aromatic changes) with deleterious functional effect, which was identified in two samples belonging to sub-lineage A.3. The residue falls directly on the ligand interaction binding site.
		Y342H (Y343H)	Amino-acid substitution (hydrophobic to moderate and neutral to positive changes) with deleterious functional effect, which was identified in several samples belonging to either sub-lineages A, B.1.1, B.1.177, B.1.258, B.1.1.284, B.1.5 or B.2.2. The residue falls directly on the ligand interaction binding site.
N	Nucleocapsid phosphoprotein	A50V	Amino-acid substitution (no amino-acid properties change) with neutral functional effect, which was identified in samples belonging to either sub-lineages B.1, B.1.1, B.1.177, B.1.80 or B.2. The residue falls on the RNA binding surface of the RNA-binding domain of N.
		R92S	Amino-acid substitution (positive to neutral change) with neutral functional effect, which was identified in a sample belonging to sub-lineage B.6. The residue falls on the RNA binding surface of the RNA-binding domain of N.

		R149L	Amino-acid substitution (hydrophilic to hydrophobic and positive to neutral changes) with deleterious functional effect, which was identified in a sample belonging to sub-lineage B.3. The residue falls on the RNA binding surface of the RNA-binding domain of N.
S	Surface glycoprotein, Spike	K417N[4]	Amino-acid substitution (positive to neutral change) with neutral functional effect, which was identified in several samples recovered mainly from patients of South Africa, belonging to lineage B.1.351 also known as 501Y.V2 [DOI: 10.1101/2020.12.21.20248640]. The residue is within the receptor-binding domain RBD.
		N439K	Amino-acid substitution (neutral to positive change) with neutral functional effect, which was identified in thousands of samples (recovered mainly from patients of Europe) belonging to either sub-lineages B.1.p73, B.1.5, B.1.1.p15, B.1.5.1, or B.1. The residue falls on the receptor-binding motif (RBM) of RBD and a SARS-CoV neutralizing antibody epitope. An equivalent position has been studied in SARS-CoV (Arg426), where at least two amino acid substitutions significantly reduced binding to ACE2. SARS-CoV-2 carrying this spike replacement exhibited immune escape from monoclonal antibodies and polyclonal sera recovered from individuals infected with variants encoding a such S mutation [DOI: /10.1101/2020.11.04.355842, DOI: 10.1016/j.cell.2020.07.012].
		F456L	Amino acid substitution (aromatic to non-aromatic ch ange) with neutral functional effect, which was identified in samples belonging to either sub-lineages B.1, B.2, or B.1.1. The residue falls on the receptor-binding motif (RBM) of RBD. An equivalent position has been studied in SARS-CoV (Leu443), where an amino acid substitution affected antibody binding and neutralization.
		G476S	Amino acid substitution (hydrophobic to hydrophilic change) with neutral functional effect, which was identified in several samples belonging to either sub-lineages A.1, B.1, B.1.1, B.1.5, B.1.p2, B.1.1.1, B.1.1.7, B.2, or B.6. The residue falls on the receptor-binding motif (RBM) of RBD. An equivalent residue has been studied in SARS-CoV (Asp463), where an amino acid substitution affected antibody binding and neutralization.
		V483A	Amino-acid substitution (no amino-acid properties change) with neutral functional effect, which was identified in several samples belonging to either sub-lineages A.1, B.1, B.1.1, or B.1.1.1. The residue falls on the receptor-binding motif (RBM) of RBD. An equivalent position has been studied in

			MERS-CoV (I529 where a single amino acid substitution increased resistance to antibody-mediated neutralization and reduced the host's receptor binding. SARS-CoV-2 carrying this spike replacement was markedly resistant to some neutralizing mAbs [DOI: 10.1016/j.cell.2020.07.012].
		E484K[4]	Amino-acid substitution (negative to positive change) with a neutral functional effect, which was identified in several samples, recovered mainly from South African patients, belonging to lineage B.1.351 also known as 501Y.V2. [DOI:10.1101/2020.12.21.20248640, https://doi.org/10.1101/2020.12.31.425021]. The residue is within the receptor-binding motif (RBM) of RBD. In SARS-CoV-2 this replacement has been associated with escape from neutralizing antibodies [DOI: 10.1016/j.chom.2020.11.007]
		N501Y[4]	Amino-acid substitution (hydrophilic to hydrophobic and non-aromatic to aromatic changes) with neutral functional effect, which was identified in thousands of samples recovered mainly from patients of both South Africa (lineage B.1.351 also known as 501Y.V2) and England (lineage B.1.1.7 of variant referred to as VOC-202012/01 on 18/12/20) [DOI: 10.1101/2020.12.21.20248640, https://doi.org/10.1101/2020.12.24.20248822]. The residue is within the receptor-binding motif (RBM) of RBD.

1 Replacement between parentheses denotes the equivalent position in the PDB: 6W9C (Nsp3), 6VWW (Nsp15). 2 Functional effects predicted by Provean algorithm (http://provean.jcvi.org). 3 Sub-lineages from CoVGLUE database (http://cov-glue.cvr.gla.ac.uk). 4. Lineages classification according to the latest pangolin version (http://pangolin.cog-uk.io/).

Table S2. Molecular weight and number of rotatable bonds of the drugs cited collected in the PubChem database. The approved drugs marked as *having active metabolites* in the e-Drug3D dataset are highlighted with an *.

Name	MW	RotB	Name	MW	RotB	Name	MW	RotB
ABARELIX	1416.06	38	ERGOTAMINE	581.7	4	NAFARELIN	1322.5	33
ADAPALENE	412.5	4	FIDAXOMICIN*	1058	15	NILOTINIB	529.5	6
ANGIOTENSIN-II	1046.2	29	FLUPHENAZINE-DECANOATE*	591.8	16	NINTEDANIB	539.6	8
BAZEDOXIFENE	470.6	7	FLUPHENAZINE-ENANTHATE*	549.7	13	OMBITASVIR	894.1	16
BETA-CAROTENE	536.9	10	GLECAPREVIR	838.9	7	PALIPERIDONE-PALMITATE*	664.9	20
BLEOMYCIN	1415.6	36	GLYCEROL-PHENYLBUTYRATE*	530.6	20	PARITAPREVIR	765.9	7
BOSUTINIB	530.4	9	GOSERELIN	1269.4	32	PASIREOTIDE	1047.2	18
CABAZITAXEL	835.9	15	GRAZOPREVIR	766.9	8	PIBRENTASVIR	1113.2	17
CABOZANTINIB	501.5	8	HISTRELIN	1323.5	34	PLICAMYCIN	1085.1	15
CALICHEAMICIN	1368.4	24	IBRUTINIB	440.5	5	POSACONAZOLE	700.8	12
CANAGLIFLOZIN	444.5	5	ICATIBANT	1304.5	30	SIMEPREVIR	749.9	8
CASPOFUNGIN	1093.3	23	IMATINIB	493.6	7	TACROLIMUS	804	7
CERULETIDE	1352.4	38	ITRACONAZOLE	705.6	11	TESTOSTERONE-CYPIONATE*	412.6	5
CETRORELIX	1491.1	38	IVERMECTIN	875.1	8	TRAMETINIB	615.4	5
CISATRACURIUM	929.1	26	LAPATINIB	581.1	11	TRIPROLIDINE	278.4	4
COBICISTAT	776	20	LEDIPASVIR	889	12	TRIPTORELIN	1311.4	33
CONIVAPTAN	535	4	LEUPROLIDE	1269.4	32	TROGLITAZONE	441.5	5
DACLATASVIR	738.9	13	LOMITAPIDE	693.7	10	VALRUBICIN	723.6	11

DACTINOMYCIN	1255.4	8	LOPINAVIR	628.8	15	VELPATASVIR	883	13
DELAVIRDINE	552.7	6	M-FIDAXOMICIN*	1058	15	VEMURAFENIB	489.9	7
DESLANOSIDE	943.1	10	MENAQUINONE	649	20	VENETOCLAX	868.4	12
DOCETAXEL	807.9	13	METOCURINE	652.8	4	VITAMIN-A-PALMITATE*	524.9	21
DOXACURIUM	1035.2	29	MIDOSTAURIN*	570.6	3	VOXILAPREVIR	868.9	9
DUTASTERIDE	528.5	2	MIVACURIUM	1029.3	30	WARFARIN	308.3	4
ELBASVIR	882	13	MONTELUKAST	586.2	12	ZAFIRLUKAST	575.7	9

Table S3. Top-20 drug candidates for repurposing for each SARS-CoV-2 according to the virtual screening experiments. Drugs currently ongoing clinical trials are highlighted in bold. The molecular weight (MW) and the number of rotatable bonds (RotB) of each compound are listed in Table S2.

PLpro		Mpro		RdRp		NendoU		Nucleocapsid		Spike	
Name	Score	Name	Score	Name	Score	Name	Score	Name	Score	Name	Score
PLICAMYCIN	-9.95	HISTRELIN	-10.71	DACTINOMYCIN	-9.88	**LOPINAVIR**	-10.12	ELBASVIR	-10.21	OMBITASVIR	-10.08
BAZEDOXIFENE	-9.65	**LEDIPASVIR**	-10.59	ABARELIX	-9.81	LOMITAPIDE	-9.95	FIDAXOMICIN	-10.00	ELBASVIR	-9.79
MENAQUINONE	-9.50	LEUPROLIDE	-10.30	ELBASVIR	-9.21	OMBITASVIR	-9.86	M-FIDAXOMICIN	-9.78	NAFARELIN	-9.78
NINTEDANIB	-9.39	ELBASVIR	-10.20	TRIPTORELIN	-9.20	VENETOCLAX	-9.86	NAFARELIN	-9.71	BETA-CAROTENE	-9.72
ZAFIRLUKAST	-9.39	NAFARELIN	-10.12	VELPATASVIR	-9.20	DOCETAXEL	-9.8	PLICAMYCIN	-9.61	PIBRENTASVIR	-9.70
DOXACURIUM	-9.24	**IMATINIB**	-10.10	PIBRENTASVIR	-9.19	VELPATASVIR	-9.73	ITRACONAZOLE	-9.60	**LEDIPASVIR**	-9.66
NILOTINIB	-9.17	PIBRENTASVIR	-9.87	**LEDIPASVIR**	-9.16	ABARELIX	-9.7	CABAZITAXEL	-9.59	VELPATASVIR	-9.56
VEMURAFENIB	-9.15	NILOTINIB	-9.85	DESLANOSIDE	-9.11	DACTINOMYCIN	-9.65	HISTRELIN	-9.57	GOSERELIN	-9.48
CABOZANTINIB	-9.13	ABARELIX	-9.85	HISTRELIN	-9.09	ELBASVIR	-9.61	NILOTINIB	-9.53	PALIPERIDONE-PALMITATE	-9.39
VITAMIN-A-PALMITATE	-9.13	CALICHEAMICIN	-9.82	CALICHEAMICIN	-9.07	LAPATINIB	-9.61	**POSACONAZOLE**	-9.51	MIVACURIUM	-9.39
ABARELIX	-9.01	CETRORELIX	-9.81	BETA-CAROTENE	-9.06	LEUPROLIDE	-9.6	**DACLATASVIR**	-9.50	PLICAMYCIN	-9.38
DELAVIRDINE	-8.98	VELPATASVIR	-9.75	DOXACURIUM	-9.04	METOCURINE	-9.58	ZAFIRLUKAST	-9.47	CETRORELIX	-9.34
CANAGLIFLOZIN	-8.98	DUTASTERIDE	-9.73	PALIPERIDONE-PALMITATE	-9.00	TRIPTORELIN	-9.58	SIMEPREVIR	-9.46	**COBICISTAT**	-9.31
PASIREOTIDE	-8.97	FLUPHENAZINE-ENANTHATE	-9.72	**ICATIBANT**	-8.97	PIBRENTASVIR	-9.56	PASIREOTIDE	-9.46	ABARELIX	-9.30
FLUPHENAZINE-DECANOATE	-8.96	ANGIOTENSIN-II	-9.69	CASPOFUNGIN	-8.95	BOSUTINIB	-9.51	GLECAPREVIR	-9.45	**MONTELUKAST**	-9.28
IBRUTINIB	-8.96	CONIVAPTAN	-9.66	GRAZOPREVIR	-8.93	MIDOSTAURIN	-9.45	**IVERMECTIN**	-9.44	DOXACURIUM	-9.27

VELPATASVIR	-8.93	OMBITASVIR	-9.64	VOXILAPREVIR	-8.93	NILOTINIB	-9.45	TRIPTORELIN	-9.44	LOMITAPIDE	-9.26
TROGLITAZONE	-8.93	**POSACONAZOLE**	-9.62	LEUPROLIDE	-8.90	TESTOSTERONE-CYPIONATE	-9.39	PIBRENTASVIR	-9.43	CERULETIDE	-9.23
ADAPALENE	-8.91	ERGOTAMINE	-9.58	MIVACURIUM	-8.89	VALRUBICIN	-9.38	VELPATASVIR	-9.55	BLEOMYCIN	-9.23
GRAZOPREVIR	-8.90	BLEOMYCIN	-9.55	VENETOCLAX	-8.89	TRAMETINIB	-9.35	**LEDIPASVIR**	-9.36	ZAFIRLUKAST	-9.22

Table S4. Top-20 scored drug candidates for repurposing against Spike wild type and each variant evaluated (*i.e.*, K417N, N439K, F456L, G476S, V483A, E484K, N501Y, and N501Y+K417N+E484K).

Rank	Wild Type	K417N	N439K	F456L	G476S	V483A	E484K	N501Y	K417N+E484K+ N501Y
1	OMBITASVIR	LEUPROLIDE	ABARELIX	HISTRELIN	CISATRACURIUM	ABARELIX	TRIPTORELIN	HISTRELIN	HISTRELIN
2	ELBASVIR	CETRORELIX	ELBASVIR	MIVACURIUM	ELBASVIR	LEDIPASVIR	CALICHEAMICIN	ABARELIX	CISATRACURIUM
3	NAFARELIN	TRIPTORELIN	MIVACURIUM	CETRORELIX	LEDIPASVIR	HISTRELIN	HISTRELIN	LEDIPASVIR	NAFARELIN
4	BETA-CAROTENE	ELBASVIR	CERULETIDE	ELBASVIR	ABARELIX	CETRORELIX	NAFARELIN	ELBASVIR	CERULETIDE
5	PIBRENTASVIR	LEDIPASVIR	DOXACURIUM	LEDIPASVIR	GOSERELIN	NAFARELIN	LEDIPASVIR	PASIREOTIDE	CETRORELIX
6	LEDIPASVIR	CISATRACURIUM	NAFARELIN	PIBRENTASVIR	NAFARELIN	ELBASVIR	CERULETIDE	CETRORELIX	PASIREOTIDE
7	VELPATASVIR	NAFARELIN	PIBRENTASVIR	GLYCEROL-PHENYLBUTYRATE	DOXACURIUM	CISATRACURIUM	GOSERELIN	DOXACURIUM	TRIPTORELIN
8	GOSERELIN	OMBITASVIR	LEDIPASVIR	DOXACURIUM	TELOTRISTAT-ETHYL	OMBITASVIR	PASIREOTIDE	CALICHEAMICIN	ELBASVIR
9	PALIPERIDONE-PALMITATE	PASIREOTIDE	TRIPTORELIN	OMBITASVIR	TRIPTORELIN	PIBRENTASVIR	MIVACURIUM	VITAMIN-A-PALMITATE	PIBRENTASVIR
10	MIVACURIUM	MIVACURIUM	HISTRELIN	CISATRACURIUM	HISTRELIN	DOXACURIUM	OMBITASVIR	OMBITASVIR	LEDIPASVIR
11	PLICAMYCIN	DACLATASVIR	DACLATASVIR	VENETOCLAX	PIBRENTASVIR	MIVACURIUM	ELBASVIR	GLYCEROL-PHENYLBUTYRATE	DOXACURIUM

12	CETRORELIX	HISTRELIN	CETRORE LIX	DACLAT ASVIR	OMBITA SVIR	GLYCER OL-PHENYL BUTYRA TE	CETRORELIX	PARITAPREVIR	MIVACURIUM
13	COBICISTAT	CERULETIDE	VENETOC LAX	ABAREL IX	MIVACU RIUM	LEUPRO LIDE	GLYCEROL-PHENYLBUTYRAT E	COBICISTAT	OMBITASVIR
14	ABARELIX	BETA-CAROTENE	CISATRAC URIUM	PLICAM YCIN	IVERME CTIN	GOSERE LIN	ABARELIX	IVERMECTIN	ABARELIX
15	MONTELUKAS T	COBICISTAT	IVERMEC TIN	VELPAT ASVIR	VENETO CLAX	BLEOM YCIN	PARITAPREVIR	DACLATASVIR	FIDAXOMICIN
16	DOXACURIUM	PARITAPREVIR	VELPATA SVIR	PASIREO TIDE	DACLAT ASVIR	IVERME CTIN	TRIPROLIDINE	MIVACURIUM	PARITAPREVIR
17	LOMITAPIDE	IVERMECTIN	ITRACON AZOLE	CERULE TIDE	CASPOF UNGIN	PASIREO TIDE	PALIPERIDONE-PALMITATE	FLUPHENAZINE-DECANOATE	LOMITAPIDE
18	CERULETIDE	PALIPERIDONE-PALMITATE	CALICHE AMICIN	CASPOF UNGIN	ITRACO NAZOLE	VELPAT ASVIR	PLICAMYCIN	TACROLIMUS	FLUPHENAZIN E-DECANOATE
19	BLEOMYCIN	VENETOCLAX	PLICAMY CIN	FIDAXO MICIN	PASIREO TIDE	VENETO CLAX	BETA-CAROTENE	FIDAXOMICIN	ZAFIRLUKAST
20	ZAFIRLUKAST	ITRACONAZOLE	FIDAXOMI CIN	CALICH EAMICI N	CALICH EAMICI N	ANGIOT ENSIN-II	WARFARIN	NAFARELIN	LEUPROLIDE

Table S5. Experimental structures of SARS-CoV-2 used in the virtual screening experiments and available at the DockThor-VS webserver.

Target	PDB code	Method	Resolution	Ligand	Reference	pH	Redocking	Grid Center X	Grid Center Y	Grid Center Z
**Nsp3 (PLpro)**	6W9C	X-ray	2.70	no	To be published	-	-	-28.532	15.815	38.918
	6WX4	X-ray	1.66	VIR251	10.1101/2020.04.29.068890	-	-			
**Nsp5 (Mpro)**	6LU7	X-ray	2.16	N3	10.1038/s41586-020-2223-y	6	-	-9.730	11.400	68.920
	6W63	X-ray	2.10	X77	To be published	-	1.338			
**Nsp12 (RdRp)**	7BV2	Electron microscopy	2.50	Remdesivir	10.1126/science.abc1560	-	-	91.760	92.370	103.720
**Nsp15 (NendoU)**	6WXC	X-ray	1.85	Tipiracil	To be published	6.2	2.612	63.945	-72.473	26.371
**N protein**	6YI3-state35	Solution NMR	-	-	To be published	-	-			
	6YI3-state12									
	6YI3-state10							15.369	-9.024	-19.694
	6YI3-state13									
	6YI3-state28									
**Spike**	6M0J	X-ray	2.45	-	10.1038/s41586-020-2180-5	-	-	-39.900	31.000	7.500
	7BZ5	X-ray	1.84	-	10.1126/science.abc2241	-	-			
